# Molecular Mechanisms in Tumorigenesis of Hepatocellular Carcinoma and in Target Treatments—An Overview

**DOI:** 10.3390/biom14060656

**Published:** 2024-06-04

**Authors:** Raluca-Margit Szilveszter, Mara Muntean, Adrian Florea

**Affiliations:** 1 Department of Pathology, Faculty of Medicine, “Iuliu Hațieganu” University of Medicine and Pharmacy, 400340 Cluj-Napoca, Romania; 2 Department of Cell and Molecular Biology, Faculty of Medicine, “Iuliu Hațieganu” University of Medicine and Pharmacy, 400349 Cluj-Napoca, Romania; muntean.mara@elearn.umfcluj.ro (M.M.); aflorea@umfcluj.ro (A.F.); 3 Cluj County Emergency Clinical Hospital, 400340 Cluj-Napoca, Romania

**Keywords:** hepatocellular carcinoma, TERT promoter mutation, p53, WNT/β-catenin, telomere shortening, histone methylation, immune checkpoint inhibitors, tumoral microenvironment, exosomes

## Abstract

Hepatocellular carcinoma is the most common primary malignancy of the liver, with hepatocellular differentiation. It is ranked sixth among the most common cancers worldwide and is the third leading cause of cancer-related deaths. The most important etiological factors discussed here are viral infection (HBV, HCV), exposure to aflatoxin B1, metabolic syndrome, and obesity (as an independent factor). Directly or indirectly, they induce chromosomal aberrations, mutations, and epigenetic changes in specific genes involved in intracellular signaling pathways, responsible for synthesis of growth factors, cell proliferation, differentiation, survival, the metastasis process (including the epithelial–mesenchymal transition and the expression of adhesion molecules), and angiogenesis. All these disrupted molecular mechanisms contribute to hepatocarcinogenesis. Furthermore, equally important is the interaction between tumor cells and the components of the tumor microenvironment: inflammatory cells and macrophages—predominantly with a pro-tumoral role—hepatic stellate cells, tumor-associated fibroblasts, cancer stem cells, extracellular vesicles, and the extracellular matrix. In this paper, we reviewed the molecular biology of hepatocellular carcinoma and the intricate mechanisms involved in hepatocarcinogenesis, and we highlighted how certain signaling pathways can be pharmacologically influenced at various levels with specific molecules. Additionally, we mentioned several examples of recent clinical trials and briefly described the current treatment protocol according to the NCCN guidelines.

## 1. Introduction—Definition, Epidemiology, and Etiology

The aim of this paper is to overview the main molecular alterations induced by etiological factors that promote the development, invasion, and metastasis of hepatocellular carcinoma (HCC) within the hepatic parenchyma. To accomplish this, we searched bibliographic sources in indexed databases (Scopus, PubMed) and selected 154 scientific works (reviews, original articles, clinical trials, treatment guidelines) that provide specific details regarding the complex molecular processes occurring in healthy hepatic parenchyma, as well as in tumor cells, and the tumor microenvironment.

HCC is a malignant proliferation of epithelial cells with hepatocellular differentiation, with the liver as its primary localization [1]. Official statistics show a global increase in the incidence of liver cancer from 2020 (ranked the seventh of all cancers [2]) to 2022 (reported in the sixth position [3]). Among the primary hepatic tumors, 75–80% are represented by HCC. It represents the third most frequent death cause linked to a malignancy in 2020 and 2022, only surpassed by pulmonary and colorectal cancer [2,3].

Chronic hepatic diseases, such as hepatitis B or C, the co-infection with both hepatitis B virus (HBV) and hepatitis C virus (HCV), or the HIV–hepatitis C co-infection [4,5,6,7,8], non-alcoholic hepatic steatosis, or steato-hepatitis [9,10] are well-known promoters of the development of HCC. Another promoter is hepatic cirrhosis, depending on its etiology: patients with hemochromatosis- or α1-antitrypsin deficit-determined cirrhosis were proven to be more susceptible than those with HBV- or HCV-induced cirrhosis [11].

Exposure to aflatoxin B1 can increase the risk of HCC in patients diagnosed with hepatitis B [12], hepatitis C, or alcohol-induced hepatitis, being described as an independent factor contributing to HCC development [13,14].

Metabolic syndrome can lead to HCC, and, in such cases, the tumors are often well-differentiated, sometimes originating in a hepatocellular adenoma, and are present in a liver with minimal fibrosis. By contrast, HCC in patients with other chronic liver diseases shows various degrees of differentiation, more severe fibrosis, and typically does not form from an adenoma [15].

There has been recent attention on the role that adipokines such as fatty acid-binding protein 4 and fatty acid-binding protein 5 (FABP4, FABP5) have in carcinogenesis [16]. Increased levels of adipokine FABP4 are associated with several metabolic conditions and significantly higher levels are found in patients with HCC and metabolic syndrome compared to healthy individuals or those with HCV-related malignancy.

Immunohistochemically, it was proven that FABP4 is expressed in endothelial cells and occasionally in the macrophages of the intratumoral sinusoids. This was replicated in vitro, using co-cultures of human umbilical vein endothelial cells (HUVEC), hepatic sinusoid endothelial cells, and HCC cell lines (HepG2, HuH7). The HUVEC cells secreted FABP4 in the culture medium in the form of microvesicles, which were subsequently captured by the HCC cells, which in turn increased viability and cell proliferation [17].

It was demonstrated that HepG2 cells also express FABP4 when exposed to fatty acids such as oleic and palmitic acid (the free fatty acids found in high concentrations in the serum of metabolic syndrome patients) [17]. An isomer of FABP4, FABP5, secreted by HCC tumor cells both in vitro and in vivo, promotes angiogenesis [18]. It was shown that FABP5 mRNA levels were much higher in the tumor, in comparison to the baseline level of the adjacent hepatic parenchyma, and the levels of FABP5 and vascular endothelial growth factor (VEGF) were also higher in the tumoral tissue when compared to the normal tissue surrounding it. Similar results were also reported for in vitro experiments: the levels of mRNA and FABP5 and VEGF were higher in the HCC cell lines in comparison to the hepatic cell line, but similarly expressed in both malignant cell lines. With an increase in the concentration of FABP5, an increase in the motility and viability of HUVEC in culture was noted, thus proving the role of this protein in angiogenesis [18].

Another research team has shown that FABP5 enhances the invasive potential, proliferation, colony formation, and metastasis ability of HCC cells, both in vitro and in vivo. FABP5 also influences the epithelial–mesenchymal transition, altering the expression of adhesion molecules and matrix-metalloproteinase 9 (MMP9), which contributes to cytoskeletal changes, basal lamina destruction, and extracellular matrix remodeling, thereby promoting metastasis [19].

Obesity is also an important factor contributing to the development and progression of HCC. Through various biological mechanisms, it induces changes in the intestinal microbiota, alterations of the tumor microenvironment, suppression of the immune response against tumors, and increased oxidative stress due to saturated fatty acids. Additionally, type 2 diabetes mellitus independently contributes to HCC tumorigenesis by increasing proinflammatory cytokines, oxidative stress, and apoptosis in hepatic cells [20].

Schlesinger et al. also revealed the higher risk of HCC development in diabetic patients that were HBV and HCV negative, independently of the duration, but associated with the early onset of the disease [21]. In developed countries, there has been a rise in the incidence of obesity and metabolic syndrome, as well as diabetes mellitus type 2, thus making the research on the secondary hepatic carcinogenesis of these pathologies (independently of chronic hepatic disease) of extreme importance, both for screening and diagnostics, as well as for the development of new anti-tumoral molecules [22,23].

Other rarer causes of HCC include Wilson disease, porphyry, hereditary thyrosinemia, and type Ia and III glycogenosis (the two latter ones being associated with the development of hepatocellular adenoma and its malignant transformation to HCC) [24].

## 2. Molecular Biology of HCC

Under the influence of the etiological factors previously described, the genetic material of hepatocytes undergoes genetic and epigenetic modifications, which play an important part in hepatic carcinogenesis [25,26].

### 2.1. Chromosomal Aberrations

Comparative genomic hybridization (CGH) data showed chromosomal aberrations in the tumor cells. Both additions (chromosomes like 1q31, 8q24.2, 6p25-p23, and 17q25), as well as deletions (chromosomes 8p21.3-p21.2, 16q, 16p13.2, 4q23-24, 17p12, and 13q21.1-q21.3) were described. In HBV-positive tumors, a higher frequency of 4q, 13q, 16q, and 8p deletions was noted, while in HCV-positive tumors, the 8p deletion was less frequent than in HCV-negative HCC. Additionally, high-resolution array-CGH data proved the existence of other alterations such as additions in the 5p15.33, 9q34.2-34.3 regions and deletions in the 6q, 9q and 14q areas [25].

Other data point out mutations in chromosomes 1, 3, 4, 6, 8, 9, 10, 11, 13, 16, and 17, such as additions or deletions, as well as the loss of heterozygosity and methylation of a cytosine–guanine site in the 3p21 region (in the gene *CTNNB1*) or the 17p13.1 region (in the gene *p53*). This goes to show the fact that altering certain chromosomal regions can lead to mutations of the genes involved in carcinogenesis [25].

### 2.2. Genetic Mutations, Altered Signaling Pathways, and Epigenetic Modifications Involved in Hepatocarcinogenesis

Tumor cells have the ability to modify their genetic and phenotypic profile through both genetic and epigenetic alterations, aiming to adapt to their environment (and to the therapeutic molecules administered). These modifications can occur in the same nodule (intratumoral heterogeneity) or in different nodules of the same patient [27,28].

The most clinically relevant mutations described in HCC cells are those of tumor suppressor genes, such as *TP53*, *ARID1/2*, telomerase-codifying genes *TERT/TERC*, as well as alterations of WNT (*CTNNB1*, *AXIN1*, *AXIN 2*) or JAK-STAT signaling pathways (genes such as *JAK1*, *IL6R*, *IL6ST*). Other signaling pathways involved in hepatocarcinogenesis are PI3K/AKT/mTOR, MYC, oxidative stress signaling pathways (with alterations of *KEAP1*, *NFE2L2* genes), RAS/RAF/MAP kinase and MET [29]. These will be detailed in the next chapters.

Signaling pathways involved in hepatocarcinogenesis can be classified as following: growth factor-dependent pathways (participating in cell differentiation), nuclear signaling pathways, and non-coding RNA-involving mechanisms (Table 1) [30]. These mechanisms will be detailed in the following sections.

### 2.3. Altered Signaling Pathways in Hepatic Carcinogenesis

**Figure 1:**
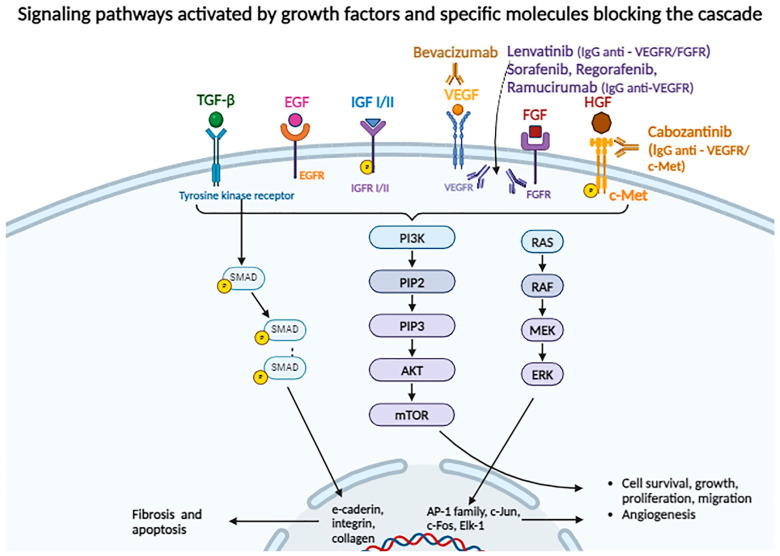
Signaling pathways activated by growth factors: the interaction of the growth factors with their specific receptors triggers the molecular cascade of PI3K/AKT/mTOR and RAS/RAF/MERK/ERK, which results in the transcription (arrow) of genes responsible for maintaining cell survival and stimulating cell proliferation, migration, and angiogenesis. TGF-β—transforming growth factor-β; EGF—epidermal growth factor; IGF I/II—insulin-like growth factor I/II; VEGF—vascular endothelial growth factor; FGF—fibroblast growth factor; HGF—hepatocyte growth factor; EGFR—epidermal growth factor receptor; IGFR I/II—insulin-like growth factor receptor I/II; VEGFR—vascular endothelial growth factor receptor; FGFR—fibroblast growth factor receptor; c-Met—mesenchymal–epithelial transition factor; SMAD—*Sma* gene and the *Drosophila* MAD (Mothers against decapentaplegic); PI3K—phosphoinositide 3-kinases; PIP2—phosphoinositide phosphatidylinositol 4,5-bisphosphate; PIP3—phosphatidylinositol-3,4,5-trisphosphate; AKT—serine/threonine-specific protein kinase (AKR mouse strain that develops spontaneous thymic lymphomas); mTOR—mammalian target of rapamycin; RAS—rat sarcoma virus; RAF—rapidly accelerated fibrosarcoma; MEK—mitogen-activated protein kinase; ERK—extracellular signal-regulated kinase; AP-1—activator protein-1; *c-Jun*—Jun proto-oncogene; *c-FOS*—a proto-oncogene that is the human homolog of the retroviral oncogene v-fos; Elk-1—ETS transcription factor ELK1.

In hepatic tumorigenesis, several stages are described. It is known that tumor cells exhibit a higher rate of replication and proliferation through certain mechanisms. On one hand, genes encoding growth factors such as IGF and FGF are overexpressed, which act in a paracrine and autocrine manner, stimulating cell multiplication. On the other hand, receptors for growth factors are overexpressed on the surface of tumor cells, leading to the persistent activation of mitogenic signaling pathways. This is the case for the c-Met receptor overexpression for HGF, which activates the Ras/Raf/MEK pathway (Figure 1).

Overexpression of the Frizzled-7 receptor leads to activation of the WNT/β-catenin pathway. Additionally, cells can interfere with the signal transduction process, thereby influencing feedback loops [26].

#### 2.3.1. FGF

FGF has been shown to participate in HCC development (Figure 1). Literature shows that the overexpression of fibroblast growth factor receptor 2 is associated with a lower degree of differentiation, worse clinical prognosis, and increased secretion of alpha-fetoprotein (AFP) [31]. Shigesawa et al. demonstrate how the cancer stem cell population is maintained by FGF2 and FGF19 [32].

#### 2.3.2. TGF

A member of this family, TGF-α is involved in the inflammatory process, regeneration, proliferation, and dysplasia [30].

In the initial stages of hepatocellular carcinogenesis, TGF-β inhibits the proliferation of premalignant hepatocytes, but, in advanced stages, under the influence of the SMAD4 protein, it becomes a proto-oncogene and contributes to the regulation of the tumor microenvironment, epithelial–mesenchymal transition, thereby promoting proliferation, migration, and invasion [30,33]. Binding of TGF-β to its specific kinase receptor can, on one hand, lead to the phosphorylation of a SMAD protein and adaptor proteins. This results in SMAD-P formation, followed by heterodimerization and translocation to the nucleus. It will further activate gene expression encoding proteins involved in fibrosis (e-cadherin, integrin, collagen), and apoptosis. On the other hand, TGF-β can activate PI3K/AKT/mTOR or RAS/RAF/MEK signaling pathways (Figure 1).

After ligand binding to the receptor, PI3K phosphorylates phosphatidylinositol 4,5-bisphosphate (PIP2) to phosphatidylinositol 3,4,5-trisphosphate (PIP3), which activates protein kinase B (AKT). This leads to the phosphorylation of cytoplasmic proteins, subsequently activating mTOR (a serine/threonine kinase) and forming complexes with other proteins, such as mTOR-mTORC1, responsible for protein and nucleotide synthesis, regulation of metabolism favoring anabolism, and cell cycle progression, stimulating cell growth and proliferation. mTORC2 induces cytoskeletal changes and activates AKT, resulting in cell survival and proliferation. PTEN is a protein that antagonizes PI3K and dephosphorylates PIP3 to PIP2 [34,35].

The RAS/RAF/MAP3K/MAP2K (MEK)/ERK pathway is responsible for cell proliferation, differentiation, angiogenesis, and survival. RAS is a GTP-binding protein on the cytoplasmic side of the cell membrane, with GDP in its inactive form; it is activated by GTP under the influence of growth factors. RAS-GTP activates RAF, which in turn phosphorylates and thus activates MAP2K-MEK1 and MEK2. MEK1 and MEK2 phosphorylate and activate ERK1 and ERK2, which translocate to the nucleus and promote the expression of genes encoding transcription factors such as *AP-1* family, *c-Jun*, *c-Fos*, and *Elk-1* gene involved in cell differentiation and proliferation. Additionally, RAS can directly bind to PI3K, activating the PI3K/AKT/mTOR pathway described above [36].

#### 2.3.3. EGF

The epidermal growth factor receptor (EGFR/ErbB1/HER1) is a membrane receptor that can bind to EGF, TGF-α, amphiregulin, epiregulin, betacellulin, and heparin-binding EGF. EGFR and its activated signaling pathways play an important role in liver tissue regeneration after acute or chronic injuries, as well as in the development of cirrhosis and HCC, where EGFR is overexpressed. Additionally, EGFR overexpression in endothelial cells plays a role in neoangiogenesis. The binding of the ligand to EGFR activates specific signaling pathways, such as Ras/Raf/MEK 1/2/ERK1/2, STAT3 and STAT5, and PI3K/AKT/mTOR (Figure 1), relevant in cellular differentiation, survival, and proliferation [37].

HER2 overexpression has been described in hepatoma cells (H4IIE, HepG2, JM1, McA-RH7777) and HCC cells collected from patients, compared to normal mature hepatocytes and cells from adjacent tumor parenchyma. In HER2-positive tumors, HER2 stimulates growth and invasion through heterodimerization with other members of the EGF receptor family and activation of the β-catenin/SMAD3 pathway. HER2 overexpression is also associated with epithelial–mesenchymal transition (EMT), the process of invasion and metastasis, as shown by Shi et al. [38].

#### 2.3.4. IGF

IGF-I and IGF-II bind to their specific receptors (IGFR-I and IGFR-II) through modulating proteins (IGFRPs) and activate the PI3K/AKT/mTOR and MAPK pathways, promoting proliferation and inhibiting apoptosis (Figure 1).

Experimental evidence has demonstrated the anti-tumor effect of inhibiting IGFR-I and, likewise, the aberrant activation of IGF-I-mediated signaling pathways as a mechanism of resistance to Sorafenib [39]. HCC consistently presents the overexpression of IGFR-I, making them sensitive to the mitogenic effect of IGF-I through autocrine mechanisms. This phenomenon has been observed both in vivo, in animal models, and in vitro, in hepatoma cell cultures. IGFBP is involved in the process of tumor invasion: the administration of IGFBP to hepatoma cell cultures (HepG2 and MHCC97-H) reduced the number of invading cells. Conversely, IGFBP gene expression became undetectable in hepatoma cells with high proliferation [40].

IGFR-2 receptor binds IGF-II, internalizes it, and undergoes lysosomal degradation, thus playing a role in modulating the signal transduction activated by IGF-II. In HCC, cells overexpress IGF-II, which correlates with reduced DNA methylation of the fetal IGF-II promoter [41].

#### 2.3.5. HGF/c-MET

Among the cells expressing the tyrosine kinase receptor c-MET are hepatocytes, epithelial cells, endothelial cells, neurons, and hematopoietic cells. HGF binding induces receptor dimerization and phosphorylation along with the phosphorylation of adaptor proteins (GRB2 and GAB1), subsequently activating signaling pathways such as PI3K/AKT/mTOR, MAPK, STAT, and NF-κB (Figure 1). Thus, HGF/c-MET plays an important role in both the initiation and progression of HCC, as well as in cell proliferation, survival, EMT, invasion, and metastasis processes [30,42].

There is a link between chronic liver diseases, HCC, and HGF/c-MET. Cirrhosis (secondary to hepatitis induced by HBV, HCV, or other etiologies) is characterized by liver regeneration, leading to the stimulation of hepatocyte proliferation through the upregulation of HGF and/or c-MET, as well as inflammation and fibrosis through c-MET activation (reducing inflammation and stimulating fibrogenesis). Although it seems to exert a beneficial effect on the progression of chronic liver pathology, c-MET can initiate the development and progression of HCC [42]. Clinical studies have demonstrated that aberrant c-MET activity is associated with tumor aggressiveness and a poorer prognosis in patients.

Increased mRNA expression, c-MET receptor overexpression, gene amplification, or other mutations in the gene responsible for c-MET transcription, elevated levels of HGF, and overexpression of receptors capable of transactivating c-MET (such as Receptor Originated from Nantes and IGFR-1) will induce altered c-MET activity. Additionally, activation of the HGF/c-MET pathway has been observed in cells treated extensively with Sorafenib, leading to increased HGF synthesis, which acts in an autocrine manner, activating c-MET and representing one of the resistance mechanisms to this agent [43].

Large tumors in particular are characterized, among other factors, by cellular hypoxia, which induces the secretion of a transcription factor, hypoxia-inducible factor-1, leading to c-MET expression. This, in turn, stimulates the expression of VEGFA, which plays a role in angiogenesis [42,44].

#### 2.3.6. VEGF

VEGF (with VEGFA as the most important isoform, followed by VEGFB, VEGFC, VEGFD, and VEGFE) and its specific receptors are important regulators of angiogenesis. Upon binding to receptors, among which VEGFR-2 is the most important, the formed complex activates Ras/Raf/MAP and PI3K/AKT/mTOR, similar to other growth factors (Figure 1). VEGF expression is stimulated by the complex resulting from the coupling of HIF with hypoxia-responsive elements (HRE), a complex influenced by the intracellular oxygen level [45].

#### 2.3.7. TP53

Normally, cell growth and proliferation are balanced through molecular mechanisms involving specific inhibitors, such as tumor suppressor proteins. One such protein is TP53. It is known that there are also regulatory proteins that ubiquitinate TP53 for degradation via the proteasome, for example, MDM2. If the gene encoding TP53 undergoes mutations (under the influence of HBV, HCV, or aflatoxin B1), or if the genes encoding MDM2 are overexpressed and amplified, there will be an imbalance in the pathway involving TP53 due to a mutant or insufficient TP53 protein, leading to uncontrolled cell proliferation. Another important aspect in this process is apoptosis. Cell death is continuously influenced by pro- and anti-apoptotic proteins. Bak and Bax are pro-apoptotic proteins that increase the permeability of the outer mitochondrial membrane for cytochrome c, which activates the caspase pathway. On the other hand, there are anti-apoptotic proteins such as Bcl-2 and Bcl-x, or regulatory proteins of the anti-apoptotic process, such as survivin. The overexpression of genes encoding Bcl-2 and survivin has been found in hepatocellular carcinoma cells [26,46].

#### 2.3.8. Cadherins

A characteristic of malignant tumors is their ability to invade adjacent tissues and to metastasize locoregionally and to other organs. This occurs partly due to alterations in tumor cells, which change from an epithelial to a fibroblastic mesenchymal-like phenotype, through the process of EMT. Additionally, it happens due to modifications in the extracellular matrix, influenced by matrix metalloproteinases, which degrade the extracellular matrix and promote invasion. EMT involves the downregulation of E-cadherin and the upregulation of N-cadherin, expressed by aggressive tumor cells with increased invasive potential [26]. E-cadherin is a transmembrane glycoprotein forming adherens junctions, composed of an extracellular segment consisting of five domain repeats, a transmembrane segment, and an intracytoplasmic segment that interacts with catenins (β-catenin, which binds p120 and α-catenin) and other proteins that attach to actin [47,48].

Through mutations or the downregulation of E-cadherin, cells lose their adhesiveness, β-catenin migrates to the nucleus where it activates the WNT/β-catenin signaling pathway, inducing the overexpression of EMT-related transcription factors such as Snail/SNAI1, Slug (SNAI2), ZEB (zinc finger E-box binding homeobox), and TWIST (twist basic helix-loop-helix transcription factor), and inhibits transcription of the *CDH1* gene (located at 16q22.1), responsible for E-cadherin synthesis [49,50,51].

Another important element is the fact that the extracellular part of E-cadherin can mainly undergo fragmentation under the influence of secretases co-catalyzed by other enzymes such as matrix metalloproteinases (MMPs: 2/3/7/9/14), disintegrins and metalloproteinases (ADAMs: 10/15/17), kallikrein 7, and plasmin, resulting in soluble E-cadherin. This presents a different oncogenic role compared to E-cadherin: it alters adherens junctions, interferes with junction formation in aggregating cells, and stimulates the activity of matrix metalloproteinases, disintegrins, and other metalloproteinases, leading to changes in extracellular matrix conformation, thereby favoring the process of tumor invasion [47,50].

#### 2.3.9. WNT/β-Catenin

**Figure 2:**
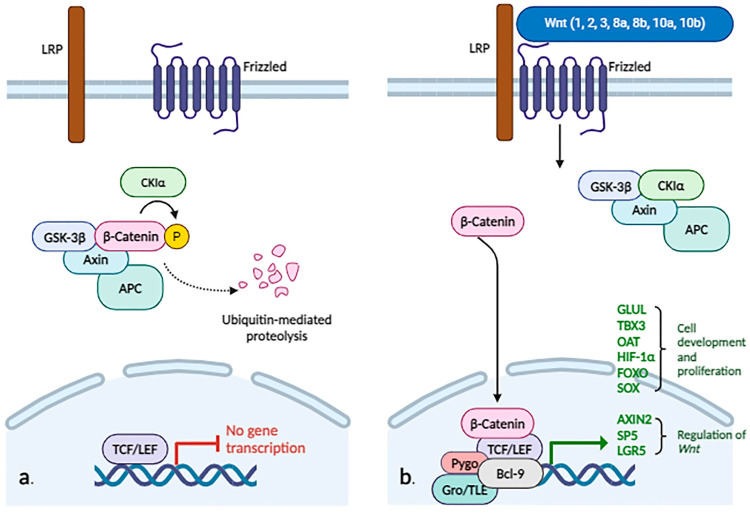
Canonical activation of the WNT/β-catenin pathway. (**a**) In the absence of WNT/β-catenin cascade activation, there is an intracytoplasmic pool of β-catenin that is phosphorylated by a protein complex, known as the “destruction complex” (formed from the GSK-3β, Axin, APC, and CKI1α proteins) that will ubiquitinate the β-catenin, undergoing proteasomal degradation. As a result, β-catenin does not interact with the transcription factors. (**b**) When the Frizzled receptor binds any member of the WNT ligand family (WNT 1, 2, 3, 8a, 8b, 10a, 10b), it activates the pathway and β-catenin is translocated to the nucleus, where it interacts with the transcription factors (TCF/LEF, Pygo, Bcl-9, Gro/TLE) that in turn trans-activate (arrow) genes known to be associated with cell development, proliferation, and WNT regulation. LRP—low-density lipoprotein receptor-related protein; WNT—Wingless/Integrated; CKIα—casein kinase Iα; GSK-3β—glycogen synthase kinase-3 beta; APC—adenomatous polyposis coli; TCF/LEF—T-cell factor/lymphoid enhancer factor; Pygo—Pygopus protein; Bcl-9—B-cell lymphoma 9 protein; Gro/TLE—*Drosophila* Groucho/transducin-like enhancers of split; GLUL—glutamate-ammonia ligase; TBX3—T-box transcription factor 3; OAT—ornithine aminotransferase; HIF-1α—hypoxia inducible factor 1 subunit alpha; FOXO—forkhead box protein O1; *SOX*—SRY-related HMG-box genes; *AXIN 2* gene; SP5—Sp5 transcription factor.

In addition to EMT, the WNT/β-catenin pathway is also involved in cellular proliferation and differentiation (Figure 2).

As is described above, β-catenin is part of the adherens junction, but it is also found in the cytoplasm and nucleus. Under normal conditions, when the WNT/β-cat pathway is not activated, β-catenin is phosphorylated by a protein complex known as the “destruction complex,” composed of the proteins AXIN, adenomatous polyposis coli (APC), glycogen synthase kinase 3 (GSK3), and casein kinase 1 (CK1).

The phosphorylated protein is then recognized by the β-transducin repeat containing E3 ubiquitin protein ligase (β-TRCP) and degraded through the proteasome, maintaining a constant cytoplasmic pool. If specific ligands of the pathway (the WNT protein family, including 19 members in humans) bind to receptors of the Frizzled (FZD) family and low-density lipoprotein receptor-related protein (LRP), β-catenin is not phosphorylated. It then accumulates in the cytosol and translocates to the nucleus, where it interacts with transcription factors (T-cell factor/lymphoid enhancer factor (TCF/LEF), Groucho/transducing-like enhancer (Gro/TLE), Pygopus (Pygo), and B-cell CLL/lymphoma 9 (BCL9)), which will trans-activate target genes such as *GLUL* (involved in cellular proliferation), *TBX3* (responsible for cellular development processes), *AXIN2* and *SP5* (regulators of the WNT pathway), *LGR5* (encodes a G-protein coupled receptor that binds R-spondins, involved in regulating the WNT pathway), *OAT* (encodes the enzyme ornithine aminotransferase, involved in the urea cycle in mitochondria), hypoxia-inducible factor 1α (*HIF1α*), forkhead box protein O (*FOXO*), and sex-determining region Y-box (*SOX*). These processes occur in the “canonical” (or β-catenin-dependent) activation of the WNT pathway [30,52,53].

Another way of activating the WNT pathway is the “non-canonical” (independent of β-catenin) pathway, which includes WNT/Ca^2+^ signaling and planar cell polarity (PCP)—these two being more studied—but also other pathways such as WNT/RYK, WNT/ROR2, WNT/casein kinase I epsilon/TERF2 interacting protein (Rap1), WNT/cyclic adenosine monophosphate/protein kinase A, WNT/DVL/atypical protein kinase C, WNT/GSK3β/microtubule, WNT/mTOR, and WNT/FYN (FYN proto-oncogene, Src family tyrosine kinase)/signal transducer and activator of transcription 3 [54]. Thus, depending on the WNT protein that binds to the receptor, canonical (WNT1, 2, 3, 8a, 8b, 10a, and 10b) or non-canonical (WNT4, 5a, 5b, 6, 7a, 7b, and 11), specific signaling pathways will be activated, dependent or not dependent on β-catenin [55].

The canonical pathway can be antagonized by ligands of the noncanonical pathway: WNT5a inhibits the activation of TCF mediated by CTNNB1, and WNT11 can, through the phosphorylation of CTNNB1, reduce the transcriptional activity mediated by TCF, thus antagonizing the canonical signaling pathway [56]. Additionally, WNT5 can interact with the Frizzled-2 receptor and activate STAT3 phosphorylation, independently of Janus kinase, a process that regulates EMT and promotes the metastasis of tumor cells [57].

A significant number of HCCs (3–44%) may present mutations in exon 3 of the gene encoding β-catenin, resulting in a mutant protein with the absence of sites that are normally phosphorylated for proteasomal degradation, resulting in chaotic pathway activation [58]. In other cases, WNT pathway initiation is due to mutations in *CTNNB1* (more frequently in HCV-related HCC), the inactivation of *AXIN1* genes (more frequently in HBV-related HCC) or *APC*, the aberrant methylation of *SOX1* and FZD related proteins, or the acetylation of secreted Frizzled-related proteins [30,59].

#### 2.3.10. JAK/STAT Pathway

**Figure 3:**
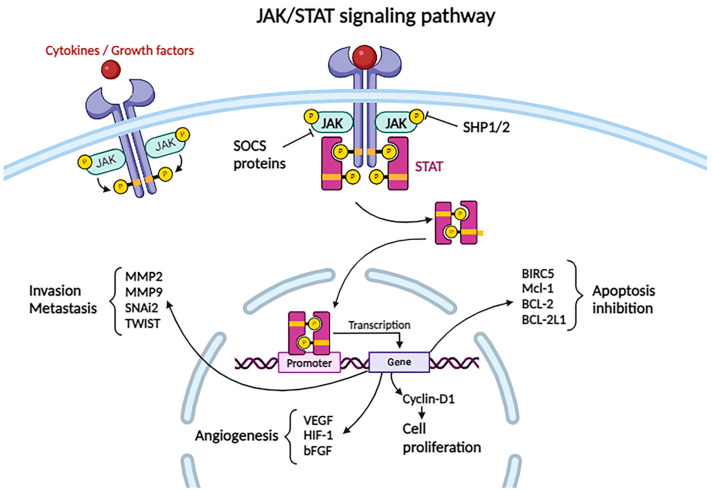
JAK/STAT signaling pathway: the activation of the JAK/STAT pathway is initiated by the binding of ligands (cytokines, growth factors) to specific receptors, associated with JAK proteins, which become activated following conformational changes triggered by the ligand. Once activated, JAK phosphorylates a tyrosine residue on the receptor, creating attachment sites for STAT that interacts with the receptor and undergoes phosphorylation by JAK, leading to the activation and dimerization of STAT proteins. The dimer is translocated to the nucleus, where it functions as a transcription factor for various genes that promote cellular proliferation, angiogenesis, tumor invasion, and metastasis. SOCs and SHP1/2 can block the cascade by competing with STAT proteins for their binding sites on the receptor or dephosphorylating JAK protein, respectively. JAK—Janus kinase; STAT—signal transducer and activator of transcription; SOCS—suppressors of cytokine signaling; SHP1/2—small heterodimer partner; BIRC5—baculoviral inhibitor of apoptosis repeat-containing 5; Mcl-1—myeloid cell leukemia-1; BCL-2—B-cell lymphoma 2; BCL2L1—B-cell lymphoma 2 like 1; VEGF—vascular endothelial growth factor; HIF-1—hypoxia inducible factor 1; MMP2—matrix metallopeptidase 2; MMP9—matrix metallopeptidase 9; SNAi2—snail family transcriptional repressor 2; TWIST—wist family bHLH transcription factor 1; bFGF—basic fibroblast growth factor.

Another important cellular signaling pathway implicated in hepatocarcinogenesis is the JAK/STAT pathway (Figure 3).

This pathway is important for regulating the immune response, hematopoiesis, and hematopoietic stem cell function, and at the hepatic level, it promotes hepatocyte proliferation, regeneration, and gluconeogenesis [60].

In humans, four members of the Janus kinase family (JAK1, JAK2, JAK3, TYK2) and seven signal transducers and activators of transcription proteins (STAT1, STAT2, STAT3, STAT4, STAT5A, STAT5B, STAT6) have been described. Depending on the ligand (usually a cytokine) that binds to the receptor, various kinds of JAK protein can be attached. From here, we understand the complexity of the cellular response when the JAK/STAT pathway is activated following receptor interaction with different ligands: for example, STAT3 and STAT5A/B are involved in tumor progression, while STAT1 exhibits a tumor-suppressive effect [61].

Activation of the JAK/STAT pathway occurs as a consequence of ligand (cytokines, growth factors) coupling to the specific receptor, which is attached to the JAK protein. The complex activates by conformational changes in the receptor and phosphorylates a tyrosine residue of the receptor, forming binding sites for STAT. STAT interacts with the receptor and will also be phosphorylated by JAK, resulting in the activation and dimerization of the STAT protein. Subsequently, the dimer is translocated to the nucleus, where it functions as a transcription factor for anti-apoptotic genes such as *CCND1*, *BIRC5*, *Mcl-1*, *BCL-2*, *BCL-2L1*, and the gene encoding cyclin D1, which is relevant in cellular proliferation. It also acts on pro-angiogenic genes such as *VEGF*, *Hif-1*, and *bFGF*, as well as on genes involved in tumor invasion and metastasis, such as *MMP2*, *MMP9*, *SNAI2*, and *TWIST1* [30,61].

The JAK/STAT pathway is regulated at three main levels: The tyrosine phosphatases SHP1 and SHP2 can dephosphorylate JAK, thereby blocking the subsequent cascade of events. The SOCS proteins (suppressors of cytokine signaling) can, on one hand, compete with STAT proteins for their binding sites on the receptor, and on the other hand, mark other proteins for proteasomal degradation. Some SOCS proteins can interact with the kinase domain of JAK, thereby inhibiting its phosphorylation. The third level at which the pathway can be inhibited is modulated by the PIAS proteins (protein inhibitors of activated STATs), which affect the binding of STAT to DNA [30,62].

#### 2.3.11. Hippo Signaling Pathway

**Figure 4:**
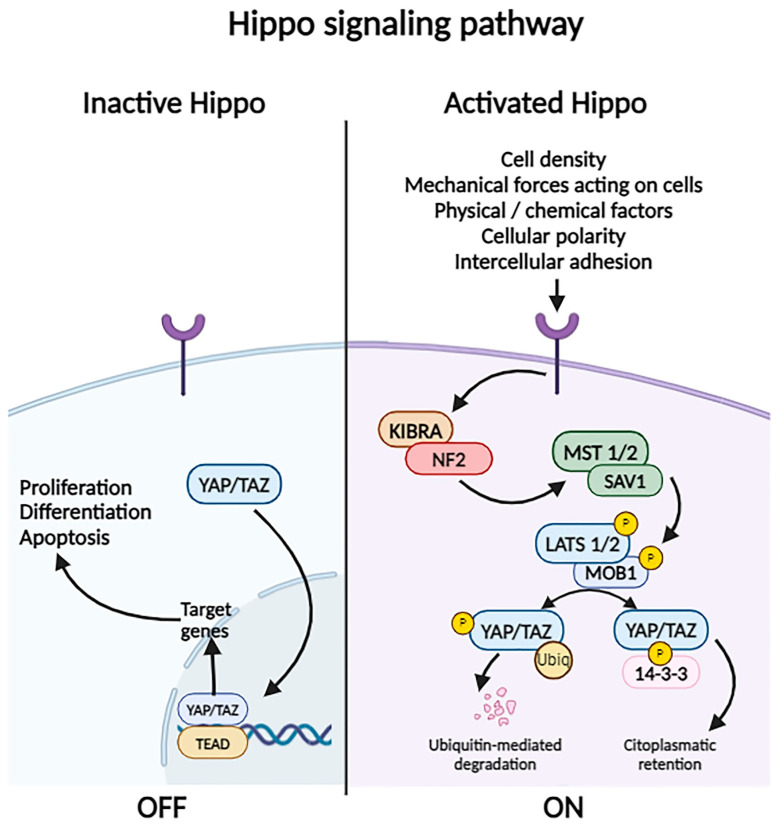
Hippo signaling pathway. When the Hippo pathway is off, YAP and TAZ proteins enter the nucleus and bind to the TEAD transcription factor, initiating the transcription of genes that promote cell survival and proliferation. Changes in the extracellular components can activate the Hippo cascade by triggering the activation of NF2 and KIBRA. These activate the kinases MST1/2, leading to the phosphorylation of the protein SAV1. The resulting complex phosphorylates the LATS1/2 and MOB by SAV1. LATS1/2 and MOB then phosphorylates YAP and TAZ. YAP/TAZ interact with the cytoplasmic protein 14-3-3, resulting in their proteasomal degradation. As a result, the transcription factor TEAD inhibits the expression of target genes. KIBRA—kidney and brain expressed protein, NF2—neurofibromatosis 2; SAV1—Salvador homolog 1; LATS1/2—large tumor suppressor homolog 1 and 2 proteins; MST1/2—monopolar spindle-one-binder proteins; YAP—Yes-associated protein; TAZ—transcriptional co-activator with PDZ-binding motif; TEAD—transcriptional enhancer activator domain; Ubiq—ubiquitin.

There are numerous factors that modulate the activation/inactivation of the Hippo pathway, such as cellular polarity, intercellular adhesion, cell density, mechanical forces acting on cells (determined by fluid mechanics, extracellular matrix components, cell geometry), and physical and chemical stress factors [63]. Changes induced in the aforementioned components, such as increased cell density, will activate transmembrane receptors that transduce the signal of neurofibromatosis 2 (NF2) and kidney and brain expressed protein (KIBRA), which in turn activate the kinases MST1/2. They phosphorylate the Salvador homolog 1 protein (WW45, the human SAV1 ortholog), which in turn phosphorylates the large tumor suppressor homolog 1 and 2 proteins (LATS1/2) and monopolar spindle-one-binder proteins (MOB). These will phosphorylate Yes-associated protein (YAP) and the transcriptional co-activator with PDZ-binding motif (TAZ), which will interact with the cytoplasmic protein 14-3-3. This leads to proteasomal degradation, so that the transcription factors TEAD1–4 will bind to the vestigial-like family member 4 protein (VGLL4) and inhibit the expression of target genes (Figure 4).

In the inactive Hippo pathway, the YAP and TAZ proteins move into the nucleus to interact with the TEAD1–4 transcription factor, activating the transcription of anti-apoptotic and cell proliferation-favoring genes [63,64,65].

As will be detailed later, the Hippo pathway can be influenced by the activation of other intracellular signaling pathways, such as WNT/β-catenin, STAT3, and Notch.

#### 2.3.12. Hedgehog (Hh) Signaling Pathway

**Figure 5:**
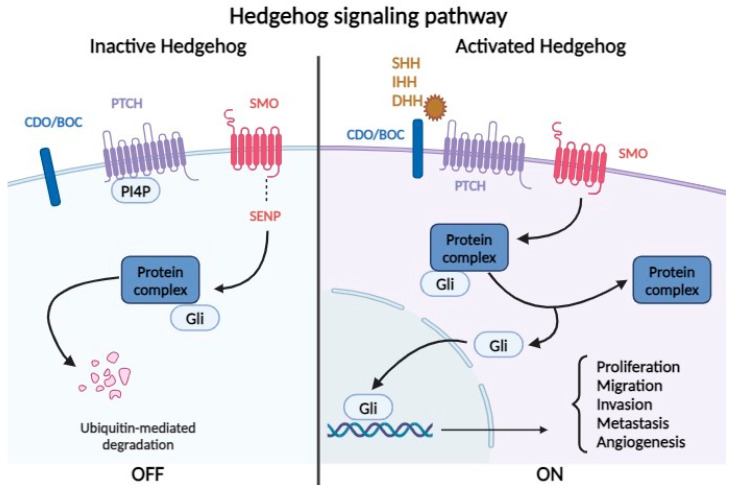
Hedgehog signaling pathway. In the absence of the binding of DHH, IHH, or SHH proteins to PTCH, phosphatidylinositol-4-phosphate PI(4)P binds to PTCH, thereby inhibiting the G protein-coupled SMO. SMO then interacts with SENP, resulting in the ubiquitination and degradation of the complex. When a ligand (DHH, IHH, SHH) binds to the CDO/BOC protein and the PTCH receptor, it triggers the activation of the G protein-coupled SMO receptor. This activation leads to the cleavage of Glis, releasing their active form from a protein complex in the cytoplasm. The activated protein is translocated in the nucleus, initiating the transcription of genes implicated in proliferation, migration, invasion, metastasis, and angiogenesis. DHH—Desert Hedgehog protein; IHH—Indian Hedgehog protein; SHH—Sonic Hedgehog protein; CDO/BOC—cysteine dioxygenase/brother of cysteine dioxygenase; PTCH—multitransmembrane protein patched; SMO—receptor smoothened; SENP—SUMO-specific peptidase protein; Glis—glioma-associated oncogene transcription factors.

The ligand family that activates this pathway includes Desert (DHH), Indian (IHH), and Sonic Hedgehog (SHH) proteins. When these proteins do not bind to the specific receptor patched protein (PTCH), phosphatidylinositol-4-phosphate interacts with it and inhibits the G protein-coupled receptor smoothened (SMO). SMO associates with the SUMO-specific peptidase protein (SENP), leading to ubiquitination and degradation of the complex. Upon ligand binding to the CDO/BOC protein and the PTCH receptor, it activates the G protein-coupled SMO receptor, which leads to the cleavage of glioma-associated oncogene transcription factors (Glis), releasing their active form from a protein complex in the cytoplasm. These proteins can translocate to the nucleus, initiating the transcription of target genes involved in cell proliferation, migration, invasion, regeneration via stem cells, and angiogenesis. Gli1 and Gli2 proteins play an activating role, while Gli3 acts as an inhibitor (Figure 5).

Aberrant activation of the Hh pathway can either occur in a ligand-dependent or an independent manner. Regardless of the ligand, this pathway can be triggered by inactivating mutations in PTCH or SMO, with the alteration of Gli protein activation and insertion of HBV in the Gli2 promoter region associated with cell proliferation. Ligand-dependent activation involves ligand overexpression—SHH protein overexpression promotes adenoma progression to hepatocellular carcinoma. Through the Hedgehog interacting protein (HHIP) receptor, the pathway can be aberrantly activated by binding DHH, IHH, and SHH ligands, reducing signaling pathway activation. Additionally, Gli1 expression promotes venous invasion and intrahepatic metastasis, and, in vitro, it stimulates cell proliferation, viability, colony formation, migration, and invasion [30,66].

Normally, the Hh signaling pathway is activated during embryogenesis, inducing the differentiation of progenitor cells from endoderm into a hepatocyte lineage. Subsequently, pathway activation occurs in response to liver parenchymal injuries [67]. For example, following partial hepatectomy, the Hh pathway is reactivated in hepatocytes, bile duct cells, and hepatic stellate cells. The latter will differentiate into myofibroblastic cells that will subsequently function as progenitors and regenerate hepatic epithelial components. However, the overexpression of SHH in stellate cells will induce the expression of pro-fibrogenic genes, and the cells will secrete collagen into the extracellular matrix.

In non-alcoholic fatty liver disease, the Hh pathway is activated depending on the ballooning of cells, inflammation, and fibrosis of the parenchyma. In the case of non-alcoholic steatohepatitis (NASH), the imbalances of the rough endoplasmic reticulum are responsible for its expansion and the accumulation of ubiquitin aggregates, giving hepatocytes a ballooned appearance (distinct from steatosis). These cells have the ability to secrete SHH and, through a paracrine mechanism, on one hand, induce pro-fibrogenic signals in neighboring hepatocytes and, on the other hand, cause a decrease in caspase-9, thereby increasing the cells’ resistance to apoptosis [68].

Activation of the Hh pathway plays a major role in hepatocarcinogenesis. Increased levels of signaling pathway components (SMO, SHH, Gli1, Gli2) have been observed in hepatocellular carcinoma cells in culture and in cells from tumors excised from patients [69]. Thus, antagonists of SMO (KAAD-cyclopamine) or anti-SHH antibodies induce the apoptosis of cells in vitro, and decreasing Gli2 expression inhibits the proliferation of hepatocytes. The role of Gli2 in the process of cellular dedifferentiation has also been described—with lower Gli2 expression in well-differentiated hepatocellular carcinoma cell lines compared to increased expression in poorly differentiated carcinoma lines [68].

#### 2.3.13. Notch Signaling Pathway

**Figure 6:**
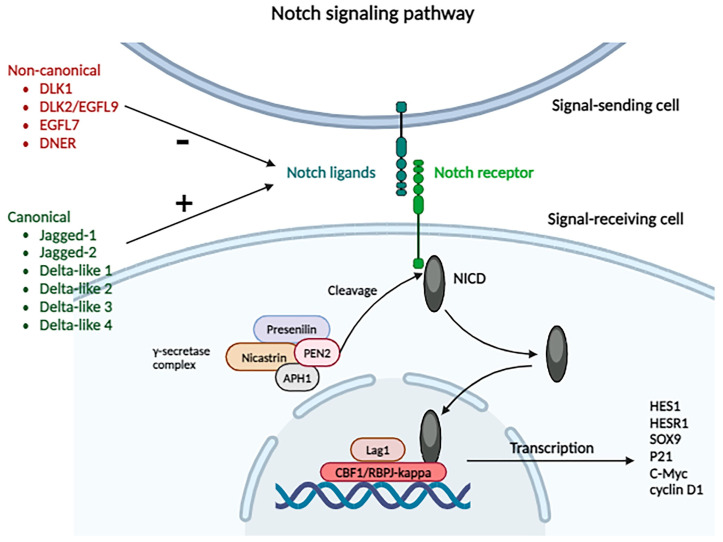
Notch signaling pathway. Upon the binding of canonical ligands to the receptor, proteolytic cleavage is triggered by the γ—secretase complex (comprising presenilin, nicastrin, APH1, and PEN2 proteins) within the receptor’s intracellular domain (NICD). This process exposes nuclear localization sequences, enabling the NICD to translocate to the nucleus. Upon coupling with proteins from the CSL (CBF1/RBPJ—kappa/Su (H)/Lag1) family, NICD acts as a transcriptional coactivator for genes belonging to the hairy and enhancer of split 1 (*HES1*), HES1—related (*HESR1*), *SOX9*, *P21*, *C—Myc*, and *cyclin D1* families, which are involved in cellular differentiation, proliferation, and apoptosis processes. DLK1—delta-like non-canonical Notch ligand 1; DLK2—delta-like non—canonical Notch ligand 2/EGFL9—EGF-like domain-containing protein 9; EGFL7—EGF—like domain-containing protein 7; DNER—delta/Notch-like epidermal growth factor (EGF)—related receptor; NICD—Notch intracellular domain; PEN2—presenilin 2; APH—anterior pharynx—defective; Lag1—longevity assurance gene 1; CBF1/RBPJ—kappa—C—repeat/DRE binding factor 1/recombination signal binding protein for immunoglobulin kappa J region.

This signaling pathway is involved in cellular differentiation and stem cell regeneration. There are four Notch receptors (Notch 1, 2, 3, 4) that can be activated by canonical ligands, such as Jagged-1, Jagged-2, and Delta-like 1, 2, 3, 4, or that can interact with non-canonical ligands, such as DLK1, DLK2/EGFL9, EGFL7, and DNER, with an inhibitory role on the pathway (Figure 6).

Activation of the pathway requires intercellular contact through the interaction of the receptor with the ligand: receptors are expressed by “receiving” cells, while ligands are expressed by signal-sending cells. Thus, after sustained interaction between the ligand and the receptor, cells change their roles: signal-sending cells express the receptor, while signal-receiving cells begin to express the ligand.

When canonical ligands bind to the receptor, proteolytic cleavage occurs under the influence of the γ-secretase complex (formed by presenilin, nicastrin, APH1, and PEN2 proteins) of the receptor’s intracellular domain (NICD). This presents nuclear localization sequences that allow its import into the nucleus, after coupling with proteins from the CSL (CBF1/RBPJ-kappa/Su (H)/Lag1) family. There, it functions as a transcriptional coactivator for genes from the hairy and enhancer of split 1 (*HES1*), and HES1-related (*HESR1*), *SOX9*, *P21*, *C-Myc*, and *cyclin D1* families, implicated in cellular differentiation, proliferation, and apoptosis processes [30]. NICD can be inhibited by phosphorylation and proteasomal degradation, or by transcriptional repression mediated by RBPJ.

Through the activation of different ligands of the four types of Notch receptors, the tumorigenesis and metastasis of HCC are favored. Research has shown elevated levels of Notch expression in tumor cells compared to the adjacent hepatic parenchyma, with variable distribution in the nucleus and cytoplasm: Notch 1 and 4 are expressed in both the nucleus and the cytoplasm, while Notch 2 and 3 are expressed only in the cytoplasm. In comparison to the adjacent parenchyma, tumor cells exhibited elevated levels of cytoplasmic Notch 1, nuclear Notch 4, and decreased levels of cytoplasmic Notch 2, with no statistically significant differences between Notch 3 and Notch 4 levels. Additionally, a correlation has been observed between increased Notch 1 expression and increased levels of AFP [70].

The Notch signaling pathway also plays an important role in angiogenesis, promoting neovascularization, smooth muscle cell differentiation, and the formation of arteriovenous structures. Notch 1 is involved in angiogenesis and vascular maturation, while Notch 3 induces smooth muscle cell maturation in vessel walls. Jagged-1 expressed by tumor cells can induce Notch pathway activation in neighboring cells; Jagged-1 is capable of binding to activated Notch 1, thereby facilitating its expression by counteracting Dll4 activity and amplifying VEGF activity. Consequently, this stimulation fosters tumor angiogenesis and facilitates tumor progression, invasion, and metastasis [70].

It has been demonstrated how the Dll4 ligand inhibits angiogenesis, in an in vitro model, using anti-Dll4 antibodies—a reduction in the size of the intratumoral vascular network was observed [71]. An increased expression of Notch 1 has been observed in advanced stage tumors with vascular invasion and lymph node metastases.

Huang et al., demonstrated how the downregulation of Notch 1 in cultured cells (MHCC97H, HepG2) reduced their invasiveness. Notch 2 was also identified at the level of hepatocellular carcinoma metastases, while Notch 4 promoted tumor aggressiveness and metastasis [70].

There have been studies demonstrating the interaction between the aforementioned signaling pathways. In tumors showing diminished activation of the Hippo pathway (abundant YAP/TAZ), the WNT/β-catenin, STAT3, and Notch pathways were activated. Notch pathway activation produced a positive feedback loop with YAP/TAZ, leading to the rapid initiation of tumorigenesis in tissues with diminished Hippo pathway activity, while β-catenin inhibited Notch, which in turn inhibited tumor initiation. Thus, activation of the Hippo pathway (which leads to YAP degradation) resulted in the decreased activation of WNT/β-catenin, Notch, and STAT3, and conversely, inactivation of the Hippo pathway activated these signaling pathways [72,73].

### 2.4. Nuclear Signaling Pathways Involved in Tumorigenesis

#### 2.4.1. Telomere Shortening and Telomerase Reactivation

Telomeres are tandem repeats of a six-nucleotide sequence of non-coding DNA (TTAGGG), located at the ends of chromosomes, protected by shelterin proteins (TRF1, TRF2, TIN2, RAP1, TPP1, and POT1). The shelterin complex attaches to the telomeres and plays a crucial role in their function. It prevents the DNA repair machinery from incorrectly identifying telomeres as double-strand DNA breaks. As telomeres shorten, DNA strand breaks occur, inducing chromosomal instability and leading to the fusion of chromosome ends if tumor suppressor mechanisms are overwhelmed. Additionally, telomere shortening triggers the activation of the ATM–ATR complex, the p53–p21 pathway, and the p16–RB1 checkpoint, ultimately resulting in cell death. Telomeres are synthesized by telomerase, which consists of telomerase reverse transcriptase (TERT), telomerase RNA component (TERC), and dyskerin. This enzyme is active during embryogenesis and in regenerative processes. Normally, telomerase activity in adult tissue cells is downregulated by silencing TERT; in adult hepatocytes and cholangiocytes, telomerase is inactive. In cells lacking telomerase, telomeres shorten, leading to their recognition as DNA damage, inducing replicative senescence, which functions as a tumor suppressor mechanism. Chronic inflammation is associated with cellular necrosis and apoptosis, thus necessitating parenchymal regeneration through hepatocyte proliferation. This eventually leads to telomere shortening over time. For example, in cirrhosis, cellular markers of senescence (p16 and β-galactosidase) have been identified, along with shorter telomeres and inactive telomerase compared to healthy hepatocytes (which exhibit low levels of senescence markers, unaltered telomeres, and the absence of telomerase activation). In an experimental model of telomerase-deficient mice with chemically and genetically induced cirrhosis, a higher rate of fibrosis was observed in comparison to control mice with normal telomerase activity [74].

It has also been observed that telomeres are shorter in tumor cells compared to adjacent hepatic cells or hepatocytes within cirrhotic parenchyma, despite elevated TERT expression in tumor cells. Thus, telomere shortening followed by telomerase reactivation is necessary for hepatocarcinogenesis. This reactivation is achieved through several mechanisms, the most common being TERT promoter mutation, observed both in tumor cells and hepatocytes within cirrhotic parenchyma [75]. The mutation of the TERT promoter is frequently associated with activating mutations of *CTNNB1*, responsible for encoding β-catenin, which induces proliferation and the development of HCC, as previously described. Another mechanism involves the insertion of HBV or adeno-associated virus type 2 into the promoter, chromosomal rearrangements, or amplifications. Through these mechanisms, TERT activity is amplified, preventing senescence by increasing cell survival and proliferation, all of which occur in the early phase of hepatocarcinogenesis.

HCV lacks the capacity for genome integration but induces chromosomal instability through chronic inflammation associated with oxidative stress, leading to mutations in various proteins such as TP53, CTNNB1, and the TERT promoter [25].

Several anti-tumor molecules targeting telomerase have been evaluated, such as Imetelstat, Telomelysin, and BIBR1532, but further testing is needed [76].

#### 2.4.2. Epigenetic Mechanisms

Epigenetics denotes heritable variations in the genome within somatic cells that cannot be ascribed to alterations in the primary DNA sequence. Epigenetic mechanisms govern chromatin conformation, nucleosome positioning, and the packaging of DNA around nucleosomes, thereby influencing the interaction of the transcriptional machinery with genes and regulating their expression. Factors impacting chromatin structure and function encompass chromatin-remodeling complexes, enzymes involved in DNA methylation/demethylation, enzymes responsible for histone modification, readers of histone marks, and non-coding RNAs [77].

##### DNA Methylation/Demethylation

The DNA molecule, by associating with eight histone proteins (two each of H2A, H2B, H3, and H4), forms nucleosomes, while, between them, Histone H1 associated with the DNA molecule compacts chromatin. Depending on the degree of compaction (condensation), chromatin is more (euchromatin) or less (heterochromatin) accessible for transcription and repair processes. However, through modifications in the structure of histones or DNA, secondary modifications of euchromatin (active) and heterochromatin (inactive) occur [77].

The most common epigenetic mechanism is the methylation of certain repetitive CpG dinucleotide sequences, which can occur frequently within the DNA, called CpG islands (5′-cytosine-phosphate-guanine-3′)—stochastic epigenetic mutations (SEMs). These CpG islands can be found in approximately 70% of gene promoters, and their methylation induces DNA condensation by inhibiting transcription initiation. Nevertheless, it has been observed that the methylation of CpG islands (CGIs) within the transcribed regions of genes leads to increased gene expression [77].

It has been demonstrated that abnormal methylation occurs at the level of HCC cells compared to adjacent parenchymal cells, with the hypomethylation (e.g., *IGF1R*, *HGF*, *HLA-DQA1*, *PIK3CG*, *MAPK10*, *KDR*, *FGFR1*, *GNGT2*, *PLCB4*, *ADCY2*), and hypermethylation of certain genes (e.g., *APC*, *HIST1H4F*, *FGF19*, *FZD1*, *LPAR2*, *HIST1H3F*, *HIST1H3G*, *HIST1H3J*, *PMAIP1*, *RASGRF2*) inducing tumorigenic metabolic alterations. The tumor grade shows a statistically significant correlation with the number of SEMs: the more epigenetic mutations cells accumulate, the higher the tumor grade and aggressiveness. Additionally, there is a link between the increased number of SEMs and viral infection (HBV, HCV, HBV + HCV) in peritumoral tissue, through direct viral action by HBV or indirectly through the chronic inflammation mediated by HCV during the methylation process [78].

Other methylated genes implicated in hepatocarcinogenesis have been described, according to Fernández-Barrena et al. [77] (Table 2).

##### Histone Modification

Histone proteins can exhibit variants or undergo post-translational modifications, such as acetylation, methylation, phosphorylation, ubiquitinylation, ADP-ribosylation, neddylation, butyrylation, succinylation, sumoylation, crotonylation, and protein glycosylation with O-linked N-acetylglucosamine. Histone variants, distinguished by one or more amino acid differences in their primary structure, exhibit specific localization and expression patterns that influence chromatin condensation and the post-translational modifications of histones. Among these, Histone H2 displays the most variants, including H2A.X, H2A.Z.1, H2A.J, H2A.Z.2.1, H2A.Z.2.2, H2A.Bbd, macroH2A1.1, macroH2A1.2, and macroH2A2. Both in vitro and in vivo studies have shown that the upregulation of H2A.Z.1 correlates with a poor prognosis. The knockdown of H2A.Z.1 in HCC cells has been found to inhibit cell growth by disrupting the transcription of cell cycle proteins and reducing metastatic potential through the selective regulation of cell cycle components. Furthermore, H2A.Z.1 is implicated in enhancing proliferation by selectively influencing regulatory proteins in the tumor microenvironment, such as upregulating E-cadherin and fibronectin [79].

All changes in the structure of histones and chromatin are mediated by enzymes that recognize target sequences (readers), others that act as transferases (writers), and enzymes that remove methyl or acetyl groups (erasers) (Table 3 [77]).

Also, the ATP-dependent chromatin remodeling complexes, which use ATP to shift nucleosomes along DNA, play significant roles in tumorigenesis. These complexes are classified into four subfamilies: SWI/SNF (switching defective/sucrose non-fermenting), ISWI (imitation SWI), NuRD/CHD (nucleosome remodeling and deacetylation/chromodomain helicase, DNA binding), and INO80 (inositol requiring 80). Research has uncovered recurrent somatic mutations in genes related to these complexes, such as *ARID1A*, *ARID2*, and *SMARCA4*, highlighting their critical involvement in the development of HCC. Additionally, the ATPase and putative DNA helicase RuvB-like 2 is often overexpressed in HCC, contributing to malignant transformation. The loss or downregulation of SWI/SNF subunits BRG1 and BRM is also common in HCC. Furthermore, the CHD family member CHD1L has been identified as having various oncogenic roles in liver cancer. These findings underscore the importance of chromatin remodeling in the progression of HCC [26].

Histone modification, such as acetylation and deacetylation, mediated by histone HATs and HDACs, respectively, influences DNA structure and subsequently regulates the accessibility of transcription factors to gene promoter regions. Among the epigenetic modifiers, HDAC deserves special attention. HDACs are enzymes that remove acetyl groups from lysine residues in the histone core, thereby influencing the transcription of certain genes by binding to other molecules with transcriptional co-repressor roles. Specific inhibitors of these enzymes are not yet approved for the treatment of HCC, but there are new promising studies on potential inhibitory molecules. Increased levels of HDAC1 and HDAC2 have been observed in patients with HCC. HDAC1 and HDAC2 levels were higher in tumoral tissue in comparison to the adjacent liver, but there was no significant difference between the intratumoral and healthy liver HDAC3 level [80]. Zhou et al. tested three distinct selective HDAC inhibitors on HCC cell lines, assessing their impact on cell proliferation and apoptosis. The results demonstrated that the selective inhibition of either HDAC1 or HDAC2 alone did not significantly influence the growth of HCC cells. However, the simultaneous inhibition of both HDAC1 and HDAC2 resulted in cell cycle arrest and apoptosis. Furthermore, the inhibition of HDAC1 and HDAC2 markedly activated the p21Waf1/Cip1 and p19INK4d signaling pathways [80]. Another study showed that the pharmacological and transcriptional inhibition of HDAC1/3 suppressed cancer cell proliferation, altered cell morphology, and downregulated the expression of HDAC1/3 in HepG2 cells: they were treated with suberoylanilide hydroxamic acid (SAHA—broad spectrum HDAC inhibitor) and romidepsin (FK228), which induced the formation of dendritic structures and triggered apoptosis at concentrations higher than 1.00 μM. Migration capacity was inhibited in HepG2 cells in a dose-dependent manner [81].

HDAC1 and HDAC2 are associated with a poor prognosis of patients. HDAC1 is frequently encountered in high-grade HCC [82]; elevated HDAC1 expression is associated with an increased incidence of cancer cell invasion into the portal vein, poorer histological differentiation, and more advanced TNM staging [83].

There are HDAC inhibitors, in addition to SAHA and FK228, which inhibit cell growth, apoptosis, cell cycle arrest, like Panobinostat, Trichostatin A, Valproic acid, Resminostat, AR-42, Droxinostat, Romidepsin, Belinostat, Quisinostat, and Pentafluorothio-substituted Vorinostat-Type histone deacetylase inhibitor [82,84].

##### Non-Coding RNAs

Non-coding RNAs (ncRNAs) are a group of RNA molecules that do not code proteins but instead play crucial roles in regulating chromatin structure and function. ncRNAs are classified into two main groups based on length: small/short ncRNAs (sncRNAs) and long ncRNAs (lncRNAs)—biomolecules formed by >200 nucleotides [79]. Small ncRNAs consist of less than 200 nucleotides and are represented by microRNAs (miRNAs), endogenous short interfering RNAs (endo-siRNAs), PIWI-interacting RNAs (piRNAs), and small nucleolar RNAs (snoRNAs). Additionally, there is the recent discovery of circular RNAs (circRNAs) as a further class of ncRNAs [79]. Out of these non-coding RNA molecules, the most investigated include miRNA, piRNA, snoRNA, circRNA, and lncRNA.

##### miRNAs

miRNAs are RNA molecules of approximately 22 nucleotides in length that function as post-translational gene regulators by binding complementarily to the protein-coding region of an mRNA molecule. Braghini et al. demonstrate in their work how, in patients with HCC, disturbances in the synthesis and export of miRNAs—specifically those suppressing oncogenes—are downregulated within the tumor microenvironment (miR-15a/16-1, miR-122, miR-139, miR-192, miR-199a/b-3p, and miR-275), whereas other miRNAs (termed “oncomiRs”) modulate genes encoding tumor suppressor proteins, thereby promoting carcinogenesis (miR-21, miR-103a, miR-221, and miR-222) [79]. In addition to tissue-specific miRNAs, circulating miRNAs are described, playing a significant role in diagnosis and patient monitoring.

Jiang et al. report that the expression of miRNA-15a-3p is downregulated in tumor tissue compared to adjacent healthy tissue, coinciding with reduced levels of the proteins HMOX1, MMP-2, CD31, and c-Myc, which consequently diminish cell proliferation, invasion, and metastasis. Moreover, in HCC cell cultures (HepG2, Huh7, Bel-7402, MHCC88H, SMMC-7221, and Hep3B), transfection with miRNA-15a-3p was observed to decrease cell viability and migratory capacity [85].

Liao et al. noted a reduced expression of miRNA-448 in tumor tissue compared to the adjacent healthy parenchyma [86]. Another study revealed that the expression levels of miR-221, miR-181b-1, miR-155-5p, miR-25, and miR-17-5p were significantly upregulated in both human and murine HCC. In Huh-7 and Hep3B HCC cell lines with stable expression of the respective miRNAs, miR-221 enhanced hepatoma cell proliferation, whereas miR-17-5p promoted cell migration [87]. Other upregulated (miR-6875-3p [88], miR-99b-3p [89]), or downregulated miRNAs (miR-218 [90], miR-320a [91], miR-17-5p [92], miR-424-5p [93]) have been mentioned in the literature.

##### piRNAs

P-Element Induced Wimpy Testis (PIWI)-interacting RNAs (piRNAs) are a class of ncRNAs that interact with PIWI proteins. These piRNAs are expressed in a tissue-specific manner across various human somatic tissues. They play crucial roles in transposon silencing, epigenetic regulation, gene and protein regulation, genome rearrangement, spermatogenesis, and the maintenance of germ stem cells. Increasing evidence indicates that the expression of several piRNAs and PIWI proteins is altered in various cancers [94].

It has been demonstrated that piR-Hep1 is upregulated in HCC cell lines compared to adjacent tissue, amplifying the PI3K/AKT signaling pathway and promoting tumor cell viability, motility, and invasion [95]. piR-017724 is found to be downregulated in HCC cell lines. Additionally, the inhibition of piR-017724 enhanced the proliferation, migration, and invasion of HCC cells. However, piR-017724 had no impact on the apoptosis of HCC cells [96]. Other piRNA molecules were shown to be upregulated (piR-28488, piR-7239, piR-5939, piR-1338, and piR-23786) or downregulated (piR-952, piR-5937, piR-5938, piR-820, piR-28525) in early HCC. Evaluating HCC tissue from advanced HCC revealed the upregulation of piR-32299, piR-23670, piR-24684, piR-28488, and piR-7239 and downregulation of piR-952, piR-820, piR-28525, piR-5938, and piR-5937 [97].

##### snoRNAs

Small nuclear RNAs (snRNAs) constitute a category of RNA molecules that reside within the nucleus. Their main role involves pre-mRNA processing, during which they consistently interact with a distinct set of proteins. These associations give rise to complexes known as small nuclear ribonucleoproteins (snRNPs). Within this classification, snoRNAs represent a subset of snRNAs located within the nucleolus. snoRNAs are intricately involved in RNA molecule maturation, primarily through chemical modifications targeted at ribosomal RNAs, transfer RNAs, and snRNAs. SNORD121B and SNORD37 were detected in early HCC tissue, while in advanced HCC, SNORD115-31, SNORD121B, SNORD121A, and SNORD37 were detected [97].

##### circRNAs

circRNA is a type of non-coding RNA that has recently garnered significant research interest. Unlike traditional linear RNA, circRNA possesses a closed circular structure, rendering it resistant to degradation by RNA exonucleases. Consequently, circRNA expression is notably stable. Recent studies have demonstrated that circRNAs, which are abundant in microRNA (miRNA) binding sites, act as miRNA sponges, sequestering miRNAs and alleviating their repressive effects on target genes. Through the competitive endogenous RNA (ceRNA) mechanism, circRNAs serve as crucial regulators in various diseases [98].

A group of scientists indicated that hsa_circ_0001306 is downregulated in HCC. hsa_circ_0001306 acts as a ceRNA, functioning as a miRNA sponge for miR-527. This interaction reduces the inhibitory effect of miR-527 on its target gene, FBXW7. Consequently, FBXW7 is upregulated in HCC, promoting cellular proliferation and invasion. FBXW7 (F-box and WD repeat domain-containing 7), a critical member of the F-box protein family, functions as a vital tumor suppressor but is inactivated in HCC. In HCC tissues, the expression of FBXW7 is significantly decreased [98]. Another study demonstrated that circular RNA circMYLK (hsa_circ_0002768) promotes the progression of HCC by functioning as a competing endogenous RNA for miR-29a, which in turn regulates the downstream oncogene KMT5C [99]. Another circRNA, hsa_circ_0091570, is also downregulated in HCC. This circRNA functions as ceRNA to inhibit HCC progression by sponging miR-1307. The reduced expression of hsa_circ_0091570 leads to elevated levels of miR-1307, which in turn decreases the expression of ISM1. This cascade promotes cell proliferation and migration while inhibiting apoptosis in HCC [99,100].

##### lncRNAs

lncRNAs are RNA molecules located in the cytoplasm, mitochondria, and nucleus, where they interact with specific proteins and mRNA, sponging and downregulating miRNA. Thus, lncRNAs mediate the apoptosis, proliferation, invasion, and metastasis of tumor cells, by modulating the activation/inhibition of cellular signaling pathways, or by interacting with the tumor microenvironment [101]. For example, DHRAS4-AS1 inhibits miR-522-3p and induces apoptosis via SOCS5 [102]; GSM3TV2 blocks miR-597 and stimulates growth and invasion by promoting FOSL2 expression [103]; FGD5-AS1 inhibits miR-873-5p, enhancing the expression of GTPBP4 [104], thereby favoring HCC progression by inhibiting miR-361-5p; SBF2-AS1 induces TGF-β1 signaling, resulting in growth and migration [104,105].

Liu et al., suggest that the overexpression of nuclear-enriched abundant transcript 1 (NEAT1) enhances HCC cell proliferation by suppressing the expression of let-7b, which is regulated by IGF-1R. The lncRNA NEAT1 exhibits aberrant expression across various human cancers, including HCC. The microRNA let-7b functions as a tumor suppressor, and its expression levels are typically reduced in tumor tissues compared to normal ones. It has been observed that IGF-1R may become abnormally activated during hepatocyte degeneration, with a notable upregulation in HCC [106]. lncRNA NEAT1 also induces EMT, thereby enhancing the invasion and metastasis of HCC cells. Studies have reported that HIF-2α upregulates NEAT1 expression, which in turn induces EMT and promotes HCC progression both in vitro and in vivo [107].

Aberrant expression of cancer susceptibility candidate 2 lncRNA has been observed in HCC. The overexpression of lncRNA upregulated in HCC influences apoptosis and cell proliferation by inhibiting the MAPK pathway [108].

Chen Wei et al., demonstrated that the downregulation of lncRNA OGFRP1 inhibited the proliferation and EMT of HCC Hep3B cells through the AKT and WNT/β-catenin signaling pathways [109]. Various other lncRNAs, such as MALAT1, SAMMSON, LINC00346, HOTAIR, TCF7, ANRIL, H19, CRNDE, SNGH5, DUXAP10, and SOX9-AS1, NEAT1, and MCM3APAS1 also regulate the WNT/β-catenin, PI3K/AKT/mTOR, and JAK/STAT signaling pathways [110,111,112,113].

Verma et al. describe in their paper that the lncRNA taurine-upregulated gene 1 promotes an increase in Hh signaling by binding to and inhibiting miR-132 from interacting with Hh, thereby contributing to HCC. Additionally, the upregulation of the lncRNAs MALAT1 and CRNDE leads to sustained activation of the Hh signaling pathway, enhancing GLI transcription factor activation and SHH associated target gene expression, which contributes to tumorigenesis in HCC [108]. Other lncRNA molecules stimulate proliferation and invasion, by activating the HGF/c-Met pathway: the elevated expression of LINC00240 in HCC leads to the sequestration of miR-4465, resulting in the upregulation of HGF with subsequent activation of the c-Met pathway [114].

lncRNAs also influence the tumoral microenvironment (described in detail in the following section). HOMER3-AS1 enhances the proliferation, migration, and invasion of HCC cells while reducing their apoptosis. Additionally, HOMER3-AS1 facilitates the recruitment of macrophages and their polarization towards an M2-like phenotype [115]. Other tumoral microenvironment cells release lncRNA in the form of small vesicles, which will support the proliferation and invasion of HCC cells [116].

According to Koduru and his team, other upregulated lncRNAs include lnc-CRK-3, GCNT1-4, HAGLR, lnc-UBC-3, lnc-TRIM27-18 (in early HCC), lnc-CCDC167-2, lnc-TPTE-3, lnc-C21orf67-10, lnc-TMEM8A-1, and lnc-CRK-3 (advanced HCC), and other downregulated ones are represented by lnc-MBNL2-3, LINC01021:16, GAS5, SNHG1:60, lnc-AC022098.1-1:10 (early HCC), lnc-SNHG6:15, HSD17B10-3, lnc-CCNB1IP1-1, GAS5, and lnc-ARHGEF6-4 (advanced HCC) [97].

ncRNAs play crucial roles in the molecular landscape of hepatocellular carcinoma, influencing a wide array of cellular processes and signaling pathways. Therefore, these molecules could serve as potential biomarkers for the diagnosis and treatment of HCC.

## 3. Tumoral Microenvironment

Another very important role in the progression of HCC is played by the interaction of tumor cells with the tumor microenvironment, represented by tumor-associated macrophages (TAMs), hepatic stellate cells, cancer-associated fibroblasts (CAFs), mesenchymal stem cells (MSCs), cancer stem cells (CSCs), endothelial cells, extracellular vesicles (including exosomes and microvesicles), and the extracellular matrix, along with the hypoxic status at this level.

### 3.1. Hypoxia

Hypoxia induces tumor angiogenesis, progression, and metastasis through the expression of VEGF and hypoxia-inducible factor 1α (HIF-1α). Members of the HIF transcription factor family (HIF-1α, HIF-2α, HIF-3α, and HIF-β) mediate the initiation of HCC (HIF-1α, HIF-2α), proliferation, and the metastatic process. Furthermore, HIF-1α and HIF-2α induce resistance to Sorafenib: after a prolonged period of administration, Sorafenib induces hypoxia in the tumor, leading to the expression of HIF-1α and NF-κB in a cellular clone more resistant to the hypoxic environment, thereby resistant to Sorafenib [117].

### 3.2. Inflammatory Cells

Immune cells actively involved in tumorigenesis include CD8+ lymphocytes, NK cells, dendritic cells, M1 macrophages with anti-tumoral roles, regulatory T cells, myeloid-derived suppressor cells (MDSCs), and M2 macrophages with pro-tumoral roles. B lymphocytes can have both anti-tumoral effects (through cytokine secretion, functioning as antigen-presenting cells alongside CD8+ cytotoxic T lymphocytes), as well as pro-tumoral effects, through cytokine secretion that attracts MDSCs and promotes angiogenesis [118]. Thus, immunotherapy is used with chemotherapy and surgical intervention.

**Figure 7:**
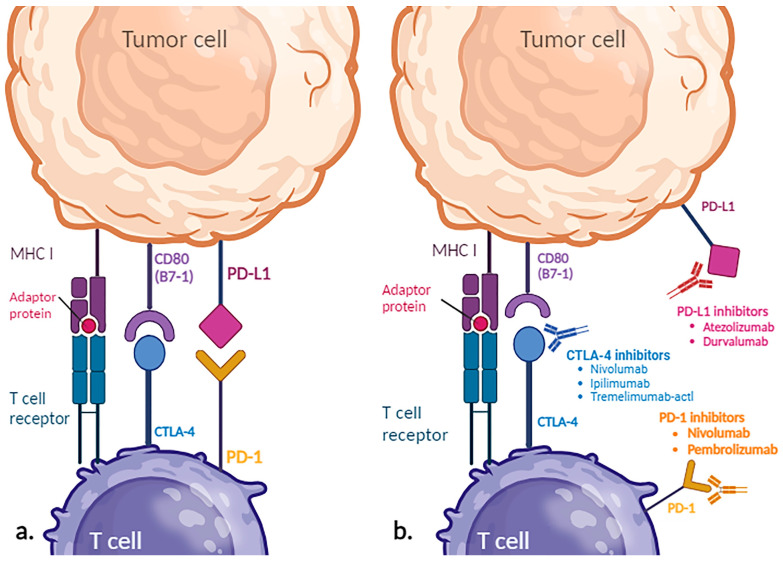
Immune checkpoints inhibitors molecular mechanism. (**a**) When PD-1 binds with PD-L1, cells are identified as self, leading to immune tolerance. Consequently, tumor cells expressing PD-L1 evade recognition by T lymphocytes. In the same way, CTLA-4 suppresses T-cell activity by binding to CD80. (**b**) Antibodies against PD-1, PD-L1, and CTLA-4, known as immune checkpoint inhibitors, will attach to the ligand/receptor and stimulate the immune response against tumor cells. MHC I—major histocompatibility complex; PD-1—programmed cell death protein 1; PD-L1—programmed cell ligand 1; CTLA-4—cytotoxic T lymphocyte antigen 4; CD80—cluster of differentiation 80.

Immunotherapic agents, such as Atezolizumab, Tremelimumab-actl, Durvalumab, Pembrolizumab, and Nivolumab, used for first-line treatment options or second-line for tumors in progression, are immune checkpoints inhibitors. Immune checkpoints are regulators of the immune response, represented by pairs of proteins (receptor–ligand) that interact and can either inhibit or activate the immune response. For example, the PD-1 receptor (Programmed Cell Death Protein 1) is expressed by T lymphocytes, while the PD-L1 ligand (Programmed Cell Death Ligand 1) is expressed by antigen-presenting cells and tumor cells (Figure 7).

Through the binding of PD-1 with PD-L1, cells are recognized as self and immune tolerance is induced. Following the PD-1–PD-L1 interaction, the proliferation of T lymphocytes is inhibited and apoptosis is induced. Through this mechanism, tumor cells expressing PD-L1 bypass immune recognition by T lymphocytes [119].

Cytotoxic T lymphocyte antigen 4 (CTLA-4), a homologue of receptor CD28, is another immune checkpoint protein that suppresses T-cell activity, by attaching to CD80 (B7-1) and CD86 (B7-2) with a higher affinity than CD28. It is primarily found on the surface of B lymphocytes, as well as activated CD8+ and CD4+ T lymphocytes. CTLA-4 is involved in regulating T-cell apoptosis, controlling cytokine expression, and suppressing T-cell proliferation, leading to an immunosuppressive effect [120]. Therefore, molecules capable of targeting PD-1 (Nivolumab and Pembrolizumab), PD-L1 (Atezolizumab, Durvalumab), and CTLA-4 (Nivolumab, Ipilimumab, Tremelimumab-actl), known as immune checkpoint inhibitors, stimulate the immune response against tumor cells [119].

In the hepatic parenchyma, in addition to resident macrophages (Kupffer cells), there are also macrophages derived from monocytes, appearing secondary to chronic inflammation or Kupffer cell destruction. Macrophages can exhibit pro-inflammatory (M1 macrophages) or anti-inflammatory (M2 macrophages) activities. M1 macrophages, activated by lipopolysaccharides or interferon-gamma (IFN-γ), secrete IL-2, which plays a role in the proliferation of effector T lymphocytes, and produce reactive oxygen species and nitric oxide, with tumoricidal roles. However, M2 macrophages, activated by IL4, IL-10, and IL-13, secrete TGF-β, IL-10, and chemokine (C-C motif) ligand (CCL) family members (CCL17, CCL18, CCL22, CCL24), with immunosuppressive effects [120].

Tumor-associated macrophages (TAMs) in HCC with activation of the WNT/β-catenin pathway induce an M2-like immunosuppressive phenotype. Through the secretion of pro-tumoral cytokines, they promote tumor development by accelerating angiogenesis and hepatocarcinogenesis. The IL-6/STAT3 signaling pathway has been demonstrated to control the polarization of M1/M2 macrophages. Inhibiting this pathway using anti-IL6 was found to decrease cell viability and drug resistance, inhibit cell invasion and migration, and trigger apoptosis in HCC cells co-cultured with M1- or M2-type macrophages. This led to the suppression of tumor formation and lung metastases [121]. A high number of TAMs is associated with large tumors, intrahepatic metastases, and a higher risk of recurrence [70].

### 3.3. Cancer-Associated Fibroblasts

With the majority of HCCs developing on a liver with cirrhosis, cancer-associated fibroblasts (CAFs) can derive from fibroblasts located within fibrous septa. CAFs promote the proliferation of tumor cells and metastasis. At the same time, HCC cells stimulate the proliferation of CAFs. These cells can secrete IL-6 which, through STAT3 phosphorylation, activates the Notch pathway in tumor cells and endows them with stem cell-like properties [70,122].

TAMs and CAFs sustain tumorigenesis, on one hand, by polarizing macrophages towards M1/M2 phenotypes (determined by Notch pathway activation), and on the other hand, by activating hepatic stellate cells involved in fibrogenesis through Notch 3 receptor activation. Hepatic stellate cells secrete collagen into the extracellular matrix and infiltrate the tumor stroma adjacent to fibrous septa and sinusoids. Furthermore, it has been demonstrated in animal models that tumor cells activate hepatic stellate cells. Thus, the interaction between TAMs and hepatic stellate cells promotes tumorigenesis by stimulating the proliferation, migration, and formation of spheroids [123].

### 3.4. Cancer Stem Cells

Cancer stem cells (CSCs) are better known as tumor initiating cells. They are highly tumorigenic, resistant to chemotherapy and radiotherapy, have a high capacity for metastasis, and can differentiate into both hepatocytic and biliary lineages, thus playing an important role in the development of HCC, intrahepatic cholangiocarcinoma, and mixed HCC–cholangiocarcinoma. Characteristically, CSCs express EpCAM, CD133, CD90, CD44, CD24, CD13, CD90, DLK1, and the oval cell marker OV6 [124].

In chronic liver disease, repeated cycles of inflammation, tissue destruction, and regeneration lead to changes in hepatic architecture and metabolism. This results in an increased number of hepatic stem progenitor cells due to the decreased regenerative capacity of hepatocytes (replicative senescence), closely correlated with the magnitude of inflammation. Changes also occur in the tumor microenvironment (as previously shown), such as genetic mutations and epigenetic alterations of cells, all favoring the induction and development of HCC. HBV infection, through DNA integration, induces changes in *p53* and *NF-κB* (resulting in tumorigenesis). HCV infection, responsible for increased oxidative stress, impacts mitochondrial metabolism. Telomere shortening, *p53* mutations induced by exposure to aflatoxin B1 and chronic inflammatory infiltrate, responsible for cytokine and chemokine release in the microenvironment, activate stellate cells and Kupffer cells (both involved in fibrogenesis). Activated myofibroblastic cells secreting growth factors, endothelial progenitor cells, and sinusoidal endothelial cells modulate angiogenesis. All these elements of the microenvironment interact with hepatic progenitor cells and hepatocytes, inducing genetic and epigenetic changes, ultimately leading to impaired hepatic regenerative processes and the development of CSCs [124].

As previously shown, HCC exhibits intratumoral (different tumor cell populations within the same tumor mass) and intertumoral (tumor cell populations within intrahepatic satellite nodules) heterogeneity, largely determined by CSC. CSCs induce HCC heterogeneity through specific surface marker expression, activating certain pathways of cellular signaling: AKT signaling for CD133+ cells, NF-κB signaling for CD24+ cells, p38 MAPK signaling for EpCAM+ cells, and NF-κB for Triple+ cells [124].

It has been demonstrated that a high number of intratumoral CSCs are associated with a worse prognosis for patients post-radical hepatic resection. However, without being able to correlate the levels of different markers expressed by CSCs with the patient prognosis, further studies are necessary [125].

### 3.5. Mesenchymal Stem Cells

Mesenchymal stem cells (MSCs) are resident cells in various organs but also integral components of the tumor microenvironment. They are represented by cells that either reside in the liver or are recruited from other tissues through chemotactic factors secreted by HCC cells or secondary to hypoxia. They are multipotent cells (able to differentiate into multiple lineages such as osteocytes, chondrocytes, and adipocytes) with a high capacity for self-renewal. Their role in the onset and progression of HCC is still intensely studied. In the early stages, they play a tumor-suppressive role, while in the advanced stages, they become tumor inductors by inducing stem-cell-like traits, promoting EMT, and increasing the number and tumorigenicity of CSCs. Additionally, through the secretion of IL-10, TGFβ, nitric acid, indoleamine 2,3 dioxygenase, prostaglandin E2, IFN-γ, and TNF-α, MSCs inhibit dendritic cells and polarize M2 macrophages. Through other secreted soluble factors, they can inhibit the proliferation of T and B lymphocytes and induce apoptosis in activated T lymphocytes. Through direct contact, MSCs inhibit the proliferation and cytokine secretion of NK T lymphocytes. Under the influence of factors secreted by tumor cells, MSCs can differentiate into cells with a CAFs-like phenotype; CAFs inhibit lymphocyte tumor infiltration, favoring immunosuppression within the tumor microenvironment [126].

The tumor-suppressive effect of MSCs on early-stage hepatocellular carcinoma tumor cells has been demonstrated, compared to their tumorigenic effect in the progressive stage of tumor development, in an experimental model of HCC induced by the oral administration of diethylnitrosamine. Concurrently, MCs were administered at 2, 4, 8, 10, and 14 weeks from the beginning of the diethylnitrosamine administration. These cells reduced the DNA structure alterations in HCC cells, as well as the number of reactive oxygen species induced by diethylnitrosamine. Additionally, the level of malondialdehyde and the total antioxidant capacity were decreased compared to the control group. In the early stage, MSCs induced anti-inflammatory and anti-fibrotic effects in the tested group compared to the control group. However, in the progressive stage, they stimulated tumor stem cells at the tumor level and the EMT process of tumor cells [127].

A group of researchers studied human amniotic MSCs morphologically, functionally, and in terms of their growth capacity and tumorigenesis in relation to hepatocellular carcinoma cells. An experimental model was established, in which BALB/c mice were subcutaneously injected with HepG2 cells, followed by the intravenous administration of human amniotic MSCs at post-injection time intervals, resulting in a reduction in tumor volume [128]. Another model used BALB/c mice into which HepG2 cells were injected in one group, and HepG2 cells + human amniotic MSCs in another. Either a small tumor nodule or no tumoral tissue could be identified in mice treated with both cell lines concurrently, compared to mice treated only with HepG2 cells. It was observed that human amniotic MSCs inhibited tumor proliferation and the apoptosis of HepG2 cells [128].

An additional study highlighted how umbilical cord MSCs co-cultured with HCC cell lines (3D culture of HCCLM3 cells) increased the viability of tumor cells but did not affect the tumor stem cells. The capacity for local invasion and metastasis, through the EMT process, was evaluated by measuring the levels of E-cadherin, N-cadherin, and vimentin. N-cadherin and vimentin were significantly increased in co-cultured cells, without changes in E-cadherin levels. The metastatic capacity was influenced by blocking the TGF-β receptor using the antagonist SB431542, resulting in increased E-cadherin levels and decreased levels of N-cadherin and vimentin in HCCLM3 cells co-cultured with umbilical cord MSCs [129].

### 3.6. Extracellular Vesicles

Extracellular vesicles, represented by exosomes, microvesicles, and apoptotic bodies, are membrane-delineated structures containing proteins (enzymes, receptors, growth factors), fragments of nucleic acids (mRNAs, miRNAs, and DNAs), metabolites, lipids, and cellular organelles belonging to the cells from which they originate (tumor cells, CAFs, CSCs). They differ in biogenesis and function. Exosomes originate from endosomes, formed by the invagination of the cell membrane, which subsequently fuse with other intracellular vesicles and lysosomes (including multivesicular bodies). The desired products to be excreted from the cell are packed into vesicles with a diameter of approximately 30–200 nm. Microvesicles result from the budding of the cell membrane and measure 0.2–1 μm in diameter. Apoptotic bodies, ranging from 50 nm to 5 μm in diameter, are formed by the blebbing and fragmentation of the cell membrane, processes that occur in apoptosis. Among these structures, exosomes are more intensively studied due to the presence of miRNA, which can be transported between tumor cells, CSCs, CAFs, and immune cells, playing an important role in the processes of tumor growth, invasion, and metastasis [130].

A significant protein involved in the formation of extracellular vesicles is Rab (member RAS oncogene family), a GTPase, whose downregulation induces the release of extracellular vesicles, thereby increasing tumorigenicity and metastatic capacity in vitro and in vivo. Another protein found in extracellular vesicles is the pyruvate kinase M2 isoform, which facilitates communication between tumor cells and monocytes/macrophages, inducing the transformation of monocytes into intratumoral macrophages and thus promoting hepatocarcinogenesis [130].

Equally important are exosomal circular RNAs, which lack a 5′-3′ end and exhibit greater chemical stability, are not degraded by exonucleases, and positively influence cellular proliferation, metastasis, and the resistance to chemotherapy [130]. In addition to circRNAs, miRNAs, long noncoding RNAs, and messenger RNAs are described, which, in most cases, induce the proliferation of tumor cells, immune evasion, and metastasis [131]. These can also exert anti-tumoral roles: for example, exosomes derived from CSCs upregulate Bax, p53, and Bcl-2, thus reducing tumor growth, progression, and metastasis. Exosomes released by CAFs induce metastasis (the loss of exo-miR-320a from exosomes or the presence of exo-miR-1247-3p is associated with metastasis to the lung). Adipocytes can release exo-miR-23a/b packed in the form of exosomes that stimulate tumor growth and metastasis. Exosomes originating from tumor cells contain exo-miR-21 and exo-miR-10b, which play roles in propagation and metastasis. Other miRNAs are involved in angiogenesis (exo-miR-21, exo-miR-26a, exo-miR-122, exo-miR-146a, exo-miR-155, and exo-miR-182), EMT (through the loss in exosomes derived from CAFs of anti-tumor exo-miR-320a), and the resistance to treatment (exo-miR-32-5p, exo-miR-744) [132]. Thus, the interaction between tumor cells and elements of the tumor microenvironment is mediated by very fine mechanisms, resulting in tumor growth, invasion, and metastasis.

## 4. Current Treatment Guidelines for HCC, According to the NCCN Guidelines

As detailed above, some signaling pathways can be pharmacologically influenced by various agents. In the following, we present the current treatment guidelines for HCC, emphasizing the mechanism of action of the various molecules recommended by the National Comprehensive Cancer Network (NCCN) [133].

Patients must meet certain criteria to undergo hepatic resection:

- A Child–Pugh score classifying them as class A or B (for highly selected patients included in class B, limited hepatic resection is appropriate);
- No portal hypertension;
- Suitable tumor location;
- Adequate liver reserve;
- Suitable liver remnant;
- Other criteria such as an AFP level ≤ 1000 ng/mL and a tumor with a diameter of 2–5 cm or 2–3 tumors with a diameter of 1–3 cm and no macrovascular involvement.

No extrahepatic diseases are considered in the therapeutic decision, i.e., in view of liver transplantation [133].

If the patient meets the criteria for resection and transplant, resection (preferably), transplant (preferably), or locoregional therapy can be considered. In case of an insufficient hepatic reserve, inadequate tumor localization, or disease extension, the patient’s case is reevaluated to assess whether they are a candidate for transplantation. If the patient is not a candidate for liver transplantation, locoregional therapy, inclusion in a clinical trial, systemic therapy (Table 4, Table 5 and Table 6), or the best supportive care are considered, depending on the tumor localization and extension, hepatic reserve, and clinical facilities available. For patients with a localized liver tumor, inoperable due to performance status, comorbidities, or with a minimal or uncertain extrahepatic tumor, locoregional therapy is preferred, along with enrollment in a clinical trial, systemic therapy, or the best supportive care. Metastatic disease or confirmed histopathologic extrahepatic extension qualifies for enrollment in a clinical trial, systemic therapy, or the best supportive care [133].

In addition to those already cited (see references), we list in Table 5 other recent clinical trials which tested different molecules, individually or in combination, alongside surgical treatment.

## 5. Concluding Remarks

HCC is a pathology with a high prevalence (among the 10 leading cancer types) and many researchers analyzing it from different points of view. The molecular biology of HCC has been extensively studied and the literature data show how complex and diverse the mechanisms through which the tumorigenesis develops are. There are several signaling pathways deciphered (in which many molecules participate), which trigger and take part in the progression of the malignant transformation.

In this paper, we have detailed hepatocarcinogenesis—a complex process involving alterations in the intracellular signaling pathways, resulting in the expression of pro-tumoral genes and the inhibition of suppressor genes, leading to cell proliferation, invasion into the adjacent hepatic parenchyma, and metastasis. Concomitant with the alteration of cellular metabolism, there is also the biogenesis of the tumoral microenvironment, through the secretion of specific molecules by tumor cells acting in an autocrine and paracrine manner. This leads to the development of cancer-associated fibroblasts and cancer stem cells from the hepatic stroma, pro-tumoral phenotypic changes in inflammatory cells (macrophages, lymphocytes), and the secretion of extracellular vesicles (by tumor cells and cells of the tumor microenvironment) supporting HCC tumorigenesis, invasion, and metastasis.

These molecular pathways also offer a high number and variety of targets for the researchers dedicated to the fight against HCC and for clinicians who use the therapies already verified or who implement the new developed treatment strategies. Thus, according to the multiple mechanisms that participate in switching the normal hepatic cells to carcinoma cells, a number of molecules have successfully been tested and are being used for the chemotherapy of HCC. We have shown how different compounds administered in systemic HCC treatment can influence the course of the disease, either by inhibiting tumor cell proliferation through blocking specific molecular pathways at various levels or by influencing elements of the tumor microenvironment. All of this diversity in molecules is important (without special mentions), since for each patient a different therapeutical approach is required.

On the other hand, the elucidation of all of these molecular mechanisms involved in the tumorigenesis of HCC facilitates the initiation of further research directions with the potential to discover other promising new ways to advance in defeating or keeping this malady under control. We are particularly interested in targeting functionalized nanoparticles with specific monoclonal antibodies against HCC in vitro and in vivo.

Another particularly important element to be highlighted is the research for new molecules and their subsequent administration to target patients. Although the new antineoplastic molecules offer numerous advantages, such as targeted therapies and prolonged patient survival, compared to existing treatments or placebo, their adoption is fraught with challenges. One significant hurdle is their high cost, which often exceeds what national health insurance programs are willing or able to cover. This financial strain is particularly problematic in countries with limited healthcare budgets. Additionally, the allocation of funds for research on these innovative molecules is another critical issue. If these research investments are not deemed cost-effective, it could hinder the development and availability of these promising therapies. Therefore, while the benefits of new antineoplastic molecules are substantial, economic and funding barriers pose considerable obstacles to their widespread implementation.

## Figures and Tables

**Table 1:** Signaling pathways involved in hepatocarcinogenesis, adapted from [30].

Growth factors and specific signaling pathway	Epidermal growth factor (EGF) and its receptor (EGFR) Fibroblast growth factor (FGF) and its receptors (FGFR) Transforming growth factor (TGF) and its receptors (TGFR) Insulin-like growth factor (IGF) and its receptors (IGFR) Hepatocyte growth factor (HGF)/c-MET RAF/ERK/MAPK PI3K-AKT-mTOR (and PTEN) VEGF and its receptor (VEGFR)
Cell differentiation and development	WNT-β-catenin JAK/STAT signaling pathway Hippo signaling pathway Hedgehog signaling pathway Notch signaling pathway
Nuclear signaling pathways	Cell cycle (TP53) Telomere shortening (TERT/TERC) Epigenetics (DNA methylation/demethylation, histone modification, non-coding RNAs)

**Table 2:** The main methylated genes relevant for hepatocarcinogenesis, adapted from [77].

Expression	Function	Gene
Hypermethylation repressed/induced	Cell cycle and cell growth regulation	APC, CDH1, CDKN1A, CDKN2A, CDKN2B, PTGS2
	Cell signaling regulation—proliferation, survival, and metastasis of HCC cells	DAB2IP, DKK,3 GNA14, HHIP, RASSF1A, SFRP2, SOCS1
	Gene transcription	ESR1, HOXA9, RUNX3, SALL3, TP73, WT1
	Metabolic regulation	CPS1, FBP1, GSTP1, IGFBP5, MAT1A
	Matrix remodeling	MMP9, MMP12
Hypomethylation induced	Gene transcription	C/EBPb
	Metabolic and signaling regulation	IGF2, NOX4, SPINK1
	Chemotaxis and angiogenesis	CCL20, ESM1

**Table 3:** Epigenetic modifiers, adapted from [77].

Readers	Writers	Erasers
MBD (methyl-CpG binding domain)-containing proteins	DNA methyltransferases	DNA demethylation system
Chromo domain-containing proteins	Histone methyltransferases: lysine	Histone demethylases
Tudor domain-containing proteins	Histone methyltransferases: protein arginine	Histone deacetylases (HDACs)
Malignant brain tumor- containing proteins	Histone acetyltransferases (HATs)	
Plant homeodomain- containing proteins	Serine-threonine and tyrosine kinases	
Bromodomain-containing proteins		
Yeats domain-containing proteins		

**Table 4:** Systemic therapy of HCC to date (adapted from [133]).

First-Line Systemic Therapy		
Options	Other recommended regimens	Useful in certain circumstances
Atezolizumab + bevacizumab or	Durvalumab	None
Tremelimumab-actl + durvalumab	Lenvatinib	
	Sorafenib	
	Pembrolizumab	
Subsequent-line systemic therapy for disease in progression		
Options	Other recommended regimens	Useful in certain circumstances
Cabozantinib	Nivolumab + Ipilimumab	Ramucirumab (AFP ≥ 400 ng/mL)
Lenvatinib	Pembrolizumab	Nivolumab
Sorafenib		Dostarlimab-gxly (for MSI-H/dMMR tumors)
		Selpercatinib (for *RET* gene fusion-positive tumors)

**Table 5:** Examples of recent clinical trials.

Treatment	Trial Phase	NCT Number	Research Team
Cabozantinib + Nivolumab	1b	NCT03299946	Ho et al., 2021 [134]
Levatinib + TACE	3	NCT03905967	Peng et al., 2023 [135]
Nivolumab	3	NCT02576509	Yau et al., 2022 [136]
Camrelizumab + Apatinib	2	NCT04297202	Xia et al., 2022 [137]
Nivolumab + Ipilimumab	1–2	NCT01658878	Yau et al., 2021 [138]
Tremelimumab + Durvalumab	1–2	NCT02519348	Kelley et al., 2021 [139]
5-Fluorouracil + Oxaliplatin hepatic arterial infusion chemotherapy	3	NCT03192618	Li et al., 2023 [140]
Tislelizumab	3	NCT03412773	Qin et. al., 2023 [141]
Pembrolizumab	2	NCT02702414	Verset et al., 2022 [142]
Lenvatinib + Toripalimab + hepatic arterial infusion chemotherapy (HAIC) with Oxaliplatin, Leucovorin, and 5-Fluorouracil (FOLFOX)	2	NCT04044313	Lai et al., 2022 [143]
Camrelizumab + Apatinib	2	NCT03463876	Xu et al., 2021 [144]
Lenvatinib + Pembrolizumab	1b	NCT03006926	Finn et al., 2020 [145]
Cadonilimab + Lenvatinib	1b-2	NCT04444167	Qiao et al., 2023 [146]

**Table 6:** Target therapies used to date for HCC.

Antineoplastic Agents	Drug	Mechanism of Action
Monoclonal antibodies	Atezolizumab	IgG1 monoclonal antibody that targets PD-L1 and inhibits the interaction between PD-L1 and its receptors, PD-1 and B7-1 (also known as CD80) [147]
	Bevacizumab	Recombinant humanized monoclonal IgG1 antibody that binds to and inhibits the biologic activity of human VEGF [147]
	Cadonilimab	Humanized, bispecific antibody against PD-1 and CTLA-4 [146]
	Camrelizumab	Humanized monoclonal IgG4-κ antibody against the PD-1 receptor [137]
	Dostarlimab-gxly	Human IgG4 monoclonal antibody against the PD-1 receptor [148]
	Durvalumab	Human monoclonal antibody (IgG1κ) that binds to the PD-L1 protein and blocks the interaction of PD-L1 with the PD-1 and CD80 proteins [149]
	Ipilimumab	Human IgG monoclonal antibody against CTLA-4 [147]
	Nivolumab	Human IgG4 monoclonal antibody against PD-1 [147]
	Pembrolizumab	PD-1 inhibitor humanized PD-1 monoclonal antibody inhibitor [132,147]
	Ramucirumab	Human IgG1 monoclonal antibody against VEGFR2 [147]
	Tislelizumab	Human monoclonal antibody directed against PD-1 [141]
	Toripalimab	Recombinant human monoclonal antibody against-PD-1 [143,150]
	Tremelimumab-actl	Human monoclonal antibody that targets the activity of cytotoxic T-lymphocyte-associated protein 4 (CTLA-4) [149,151]
Protein kinase inhibitors	Apatinib	Tyrosine kinase inhibitor that selectively inhibits VEGFR2 [137]
	Cabozantinib	A receptor tyrosine kinase inhibitor that acts on MET, VEGFR, RET, AXL (GAS6 receptor), KIT, and FLT3 [147]
	Lenvatinib	Multi-kinase inhibitor that inhibits VEGFR1–3 as well as FGF receptors 1–4 along with platelet-derived growth factor receptor (PDGFR), c-KIT [147]
	Regorafenib	Multi-kinase inhibitor with anti-angiogenic activity against VEGFR2 [149]
	Selpercatinib	Inhibitor of tyrosine kinase receptor encoded by the RET gene (rearranged during transfection) [152]
	Sorafenib	Inhibitor of the serine-threonine kinases Raf-1 and B-Raf and the receptor tyrosine kinase activity of VEGF receptors 1, 2, and 3 and PDGFR-β [153]
Others	5-Fluorouracil (5-FU)	Heterocyclic aromatic organic compound which interferes with nucleoside metabolism and can be incorporated into RNA and DNA, leading to cytotoxicity and cell death [140,154]
	Oxaliplatin	Oxalato(trans-l-1,2-diaminocyclohexane) platinum that induces apoptosis by DNA damage, blocking DNA synthesis, the suppression of RNA synthesis, and the initiation of immune responses [140]
